# Pulmonary Proteomic Responses to Subacute 2,2′,5,5′-Tetrachlorobiphenyl-4-ol Exposure via a Subcutaneous Implant in Adolescent Female Rats

**DOI:** 10.3390/jox16060189

**Published:** 2026-10-06

**Authors:** Crystal M. Roach, Hui Wang, Nate R. Koester, Joel D. Nott, Xueshu Li, Hans-Joachim Lehmler

**Affiliations:** 1 Department of Occupational and Environmental Health, College of Public Health, The University of Iowa, Iowa City, IA 52242, USA; crystal-roach@uiowa.edu (C.M.R.); hui-wang-1@uiowa.edu (H.W.); nathan-koester@uiowa.edu (N.R.K.); xueshu-li@uiowa.edu (X.L.); 2 Protein Facility, Office of Biotechnology, Iowa State University, Ames, IA 50011-1079, USA; protein@iastate.edu

**Keywords:** adolescent rats, female, lung, polychlorinated biphenyls, polymeric implant, proteomics, 4-OH-PCB52

## Abstract

Hydroxylated polychlorinated biphenyls (OH-PCBs) are biologically active metabolites that may contribute to adverse respiratory outcomes, yet their effects on the developing lung remain poorly characterized. This study investigated the impact of 2,2′,5,5′-tetrachlorobiphenyl-4-ol (4-OH-PCB52), a human-relevant metabolite, on the pulmonary molecular pathways in postnatal day 28 female adolescent rats using a multi-omics approach. Animals were exposed to 4-OH-PCB52 (sham; low 1%; medium 5%; high 10%; (*w*/*w*)) via a subcutaneous implant release system for 28 days. Targeted analysis by gas chromatography–tandem mass spectrometry (GC-MS/MS) detected 4-OH-PCB52 in the medium 5% and high 10% exposure dose groups. At both higher doses, proteomic profiling revealed significant alterations in protein markers related to pulmonary post-transcriptional regulation, metabolism and chemical biotransformation, immunity and inflammation, oxidative stress and programmed cell death, and surfactant production and mucociliary clearance. In contrast, RNA sequencing did not identify differentially expressed genes. Collectively, these findings demonstrate that adolescent exposure to 4-OH-PCB52 perturbs key molecular markers associated with lung homeostasis, with potential implications for altered lung development and increased susceptibility to respiratory disorders during adulthood.

## 1. Introduction

Polychlorinated biphenyls (PCBs) are organochlorine compounds that persist in the environment and continue to pose significant public health risks, despite the prohibition of their production in the United States in the late 1970s. PCBs were widely produced for a variety of industrial applications, including use as caulking compounds, coolants, electrical insulators, flame retardants, and lubricants [1]. PCBs originate from both intentional (legacy) and unintentional (nonlegacy) production, with the latter arising as byproducts during the manufacture of pigments and dyes [2] and silicone materials [3]. Due to their high lipophilicity and resistance to environmental and biological degradation, PCBs readily bioaccumulate in organisms and biomagnify through the food chain [4]. Consequently, human exposure remains widespread, occurring primarily through dietary intake of contaminated food and inhalation of indoor and outdoor air [5].

Humans are exposed to lower-chlorinated PCBs (LC-PCBs; ≤ 4 chlorine substituents) through dietary intake, inhalation, and dermal contact [6]. Among these, 2,2′,5,5′-tetrachlorobiphenyl (PCB52) is a major component of commercial PCB mixtures. Toxic effects of PCB52 have been reported to occur independently of activation of the aryl hydrocarbon receptor (AhR), an important cellular target of PCBs [7], and have been widely reported in environmental and biological samples [5,8,9,10]. PCB52 undergoes biotransformation in rodents and humans to hydroxylated metabolites, including 2,2′,5,5′-tetrachlorobiphenyl-3-ol (3-OH-PCB52), 2,2′,5,5′-tetrachlorobiphenyl-4-ol (4-OH-PCB52), and 3,4-dihydroxy-2,2′,5,5′-tetrachloro-biphenyl (3,4-diOH-PCB52) [11,12,13]. 4-OH-PCB52 is the only PCB52 metabolite formed by human cytochrome P450 enzymes, specifically CYP2A6 [14]. During PCB52 metabolism, hydroxylated metabolites such as 4-OH-PCB52 can undergo further oxidation to form quinone metabolites. These PCB52 quinones, a class of Michael acceptors, react with DNA, lipids, and proteins, promoting oxidative stress and contributing to oxidative DNA damage [15].

Exposure to PCBs has been associated, in humans and animals, with multisystem toxicity, including carcinogenesis [16], endocrine and metabolic disorders [17,18], immune dysfunction [19], and developmental and reproductive effects [20,21]. However, comparatively few studies have examined the effects of LC-PCBs and their metabolites on the lung as a target organ. Allergenic diseases, including asthma, represent a major global health concern and are among the most common noncommunicable diseases in children [22]. These conditions often originate in childhood and can continue into adulthood. Adolescents are particularly susceptible to PCB-associated adverse health effects, as the respiratory and immune systems continue to mature during this developmental period [23]. Growing evidence suggests that PCBs may contribute to the risk of allergic and respiratory diseases, with several studies reporting associations between early-life PCB exposure and childhood asthma or related respiratory symptoms [24,25]. Notably, higher blood levels of LC-PCBs in children aged 3–6 years have been associated with increased risk of childhood asthma [26].

While these studies support a link between PCB exposure and lung-related adverse health outcomes, the effects of OH-PCB metabolites on the lung remain poorly defined due to species-dependent differences in PCB metabolism and the limitations of traditional exposure paradigms. Consequently, experimental approaches that enable controlled, sustained delivery of individual, human-relevant PCB metabolites are needed to better characterize their biological effects. Polymeric implants offer a minimally invasive method for sustained metabolite delivery that may better model subacute human exposure [27], as other traditional methods can be invasive, induce stress responses, or subject compounds to hepatic first-pass metabolism [28,29]. Leveraging this approach, we previously demonstrated that 4-OH-PCB52 is released from the implant and distributed to target tissues, resulting in molecular alterations in adolescent male rats [30,31].

Here, we collected and processed lung tissues from adolescent female rats exposed to 4-OH-PCB52, a human metabolite of PCB52, delivered via a subcutaneous polymeric implant to investigate the molecular mechanisms underlying pulmonary toxicity. The study of adolescent females is particularly important because sex-specific differences in immune function, hormone signaling, and toxicant responses may influence susceptibility to PCB-induced respiratory effects [32,33]. In this exploratory study, we evaluated the sustained release of 4-OH-PCB52 from the implants, quantified PCB metabolite disposition, and characterized pulmonary transcriptomic and proteomic changes. Our findings identify molecular changes associated with 4-OH-PCB52 exposure and highlight potential biomarkers and pathways that may contribute to hydroxylated PCB-induced effects in adolescent female rats.

## 2. Materials and Methods

### 2.1. Chemicals and Analytical Standards

Analytical OH-PCB standards for GC-MS/MS analysis, including 2,2′,5,5′-tetrachlorobiphenyl-4-ol (4-OH-PCB52) and 4,4′-dimethoxy-2,2′,5,5′-tetrachlorobiphenyl (methylated standard for 4,4′-dihydroxy-2,2′,5,5′-tetrachlorobiphenyl; 4,4′-diOH-PCB52), were synthesized and authenticated as described [30]. The surrogate standard, 2,4′,5-trichlorobiphenyl-4-ol (4-OH-PCB25), and internal standards, 2,4,6-trichlorobiphenyl (PCB30) and 2,2′,3,4,4′,5,6,6′-octachlorobiphenyl (PCB204), were purchased from AccuStandard (New Haven, CT, USA). Diazomethane was synthesized from N-methyl-N-nitroso-p-toluenesulfonamide (MilliporeSigma, Burlington, MA, USA) using a mini Diazald apparatus (MilliporeSigma) [34,35]. Additional information regarding analytical standards is available in the Supplementary Materials, Table S1. All chemicals and pesticide-grade solvents (dichloromethane, hexane, isopropanol, methanol), concentrated hydrochloric acid (HCl), concentrated sulfuric acid, potassium chloride, and silica gel (0.062–0.105 mm, 150–230 mesh) for GC-MS/MS were purchased from Thermo Fisher Scientific (Waltham, MA, USA).

### 2.2. Animal Exposure and Experimental Design

The animal experiment was approved by the Institutional Animal Care and Use Committee (IACUC) guidelines under Protocol No. 0101265, approved on 6 January 2021 at the University of Iowa and conducted in compliance with the NIH National Research Council’s *Guide for the Care and Use of Laboratory Animals*. A total of sixty female juvenile Sprague–Dawley rats (postnatal day 21; PND 21), weighing 50 ± 4 g, were obtained from Envigo (Indianapolis, IN, USA). The animal numbers were determined by a previously published male polymeric implant study [30] to have sufficient power to detect differences between exposure groups in neurobehavioral outcomes. For logistical scheduling, animals were divided into four cohorts (*n* = 15 per cohort), staggered by 3 weeks. Rats were housed in fiber-covered polypropylene cages within an AAALAC-accredited facility maintained at 20–26 °C, 55% relative humidity, and a 12 h light:dark cycle. A standard chow diet (sterile Teklad 5% stock diet; Harlan, Madison, WI, USA) and water were provided *ad libitum*.

Following a seven-day acclimation period, rats (PND 28) were randomly assigned (via Random.org) to sham, low-, medium-, or high-dose exposure groups (*n* = 15 per exposure group). Polymeric implants containing 0, 1, 5, or 10% 4-OH-PCB52 (~1.2 ± 0.2 mg/g body weight [bw]; *w*/*w*) were surgically implanted subcutaneously in the upper back on PND 28 and maintained in situ for 28 days, until PND 56. Detailed procedures for polymeric implant surgery have been previously published [30]. Animals were monitored daily. Body weights were recorded twice a week. On PND 56, rats were euthanized via isoflurane inhalation followed by cardiac puncture, and tissues were immediately collected and snap-frozen at −80 °C for subsequent analysis.

Histopathological evaluation of the accessory lobe of the right lung was performed by a board-certified veterinary pathologist at Iowa State University’s Comparative Pathology Core Service (Ames, IA). Two animals were randomly selected from each cohort, resulting in a total of 8 animals per exposure group. Hematoxylin and eosin (H&E)-stained lung sections were examined for evidence of inflammation, hemorrhage, edema, necrosis, apoptosis, extramedullary hematopoiesis, and other morphological alterations. Inflammation was defined by the presence of infiltrating immune cells.

Residual 4-OH-PCB52 content in the implants on PND 56 was quantified spectrophotometrically. Internal dose was estimated by subtracting the residual amount from the initial loading, assuming complete bioavailability of the 4-OH-PCB52 released from the implant. Human-equivalent oral intake and lifetime inhalation exposure, as well as polymeric implant design, have been described previously [30]. No adverse effects were observed during the exposure period.

Experimental samples from 1–2 rats per exposure group per cohort were randomly selected for further analysis. No a priori sample size calculation or statistical power analysis was performed, as no single primary endpoint was defined for this purpose; as such, this study should be considered exploratory in nature. Instead, the sample size was selected based on tandem mass tagged (TMT) workflow. Because each TMT batch could accommodate ten biological samples, a target sample size of 20 animals was selected, allowing all samples to be analyzed in only two TMT batches. One sample from the low-dose group did not meet the quality control standards and was excluded from further analysis. This yielded a final sample size of *n* = 19 (sham: *n* = 5; low 1%: *n* = 4; medium 5%: *n* = 5; high 10%: *n* = 5). Investigators were aware of group allocation during sample runs, as samples were to be processed in order of increasing dosage.

### 2.3. Targeted 4-OH-PCB52 Analysis

#### 2.3.1. Extraction of 4-OH-PCB52

Lung tissues (*n* = 19; 0.099 ± 0.007 g) were homogenized in isopropanol (3 mL), after which diethyl ether (1 mL) was added. Each sample was spiked with a surrogate recovery standard (10 ng; 4-OH-PCB25, 100 ng/mL in methanol). For ongoing precision and recovery (OPR) and reference samples, analytes (4-OH-PCB52, 100 ng/mL in methanol; 4,4′-diOMe-PCB52, 100 ng/mL in isooctane containing 10% toluene) were added at 10 ng each. Samples were mixed by inversion for 5 min at 40 rpm and centrifuged at d*g* for 5 min. The organic phase was transferred to a fresh tube containing phosphoric acid (5 mL; 0.1 M in 0.9% sodium chloride). Isopropanol was added (1 mL), and tissue pellets were resuspended in hexane:diethyl ether (3 mL; 9:1, *v*/*v*), vortexed for 10 s, and centrifuged at 1811 *g* for 5 min. Organic phases were combined, mixed by inversion, and centrifuged again at 1811 *g* for 5 min. The resulting organic extract was moved to a clean glass tube, and the aqueous phase was re-extracted with hexane:diethyl ether (3 mL; 9:1, *v*/*v*). Combined organic extracts were evaporated to ~100 µL under a slow stream of nitrogen.

To methylate hydroxylated analytes, five drops of methanol were added and vortexed, followed by diazomethane in diethyl ether (0.5 mL; 0.12 mmol). Extracts and reference samples were stored at 4 °C overnight (~16 h). Excess solvent and diazomethane were removed under a gentle stream of nitrogen to ~200 µL. Reference samples were concentrated to ~50 µL and reserved for internal standard spiking.

Clean-up was performed using solid-phase extraction cartridges packed with glass wool (bottom), activated silica gel (0.2 g; middle), and acidified silica gel (2 g; silica gel: H_2_SO_4_, 5:1, *w*/*w*; top). Columns were preconditioned with hexane:dichloromethane (3 mL; 1:1, *v*/*v*). PCBs and OH-PCBs (as methylated derivatives) were eluted with hexane:dichloromethane (10 mL, 1:1, *v*/*v*) and concentrated to ~200 µL under nitrogen gas. Hexane (3 mL) was added and further concentrated to ~50 µL. Extracts and reference samples were spiked with internal standards (10 ng each; PCB30 and PCB204 in isooctane) and transferred to GC vials for GC-MS/MS analysis.

#### 2.3.2. GC-MS/MS

Instrumental analyses were performed on an Agilent 8890A gas chromatograph coupled to an Agilent 7000E Triple Quadrupole Mass Spectrometer (Agilent Technologies, Santa Clara, CA, USA), operated in multiple reaction monitoring (MRM) mode to quantify target analytes and surrogate standards. Chromatographic separation was achieved using an SPB-Octyl capillary column (50% n-octyl/50% methyl siloxane, 30 m length, 250 µm inner diameter, 0.25 µm film thickness; MilliporeSigma). Helium served as the carrier gas at a constant flow rate of 0.8 mL/min, and nitrogen was used as the collision gas.

Samples were injected through a temperature-programmed split/splitless injector, beginning at 50 °C with a 0.06 min hold, followed by a ramp to 325 °C at 600 °C/min and a 10 min hold. The GC oven program started with a 2 min hold at 50 °C, followed by a ramp to 75 °C at 100 °C/min with a 3 min hold, then increased to 170 °C at 100 °C/min, and finally to 280 °C at 2.5 °C/min with a 5 min hold. The MS transfer line was maintained at 280 °C. Transitions for precursor ions, product ions, and collision energies are provided in Supplementary Materials, Table S2.

#### 2.3.3. Quality Assurance and Quality Control (QA/QC)

GC-MS/MS analyses incorporated solvent blanks, method blanks, vehicle-exposed animal tissues, and OPR samples analyzed concurrently with study samples to verify analytical performance and method consistency. Neither 4-OH-PCB52 nor dihydroxylated metabolites were detected in any blank matrices. Summary statistics for OPR performance, including mean recoveries, recovery ranges, and relative standard deviations by analyte, along with surrogate recoveries, method detection limits (MDL), limit of detection (LOD), and quantified concentrations (ng/g) of 4-OH-PCB52 and metabolites, are reported in Supplementary Materials, Tables S3–S6.

### 2.4. Transcriptomics

#### 2.4.1. RNA Extraction and Sequencing

Total RNA was extracted from the lung tissue of the same 19 rats used in the proteomics analysis with the QIAGEN RNeasy Kit (QIAGEN, Hilden, Germany) following the manufacturer’s protocol. RNA quantity and purity were quantified with a NanoDrop spectrophotometer (Thermo Fisher Scientific), with all samples meeting quality thresholds (A260/280 = 1.8–2.2; A260/230 ≥ 1.8). Samples were submitted to Novogene Corporation Inc. (Sacramento, CA, USA) for mRNA sequencing. RNA integrity was measured with an Agilent 2100 Bioanalyzer (Agilent Technologies, Santa Clara, CA, USA), yielding 18 samples with an RNA integrity number (RIN) ≥ 7.0; one was excluded. cDNA libraries were prepared after ribosomal RNA depletion and sequenced as 120-bp paired-end reads on an Illumina platform using the Illumina 1.9 sequencing protocol, in accordance with Novogene procedures.

#### 2.4.2. RNA-Seq Data Processing and Bioinformatic Analysis

Raw sequence data (FASTQ files) were demultiplexed and concatenated per sample, followed by quality assessment with FastQC (v0.11.9). High-quality reads were then aligned to the *Rattus norvegicus* reference genome (UCSC rn6) using HISAT2, achieving above 86% mapping efficiency across all samples. Resulting Sequence Alignment/Map (SAM) files were converted to sorted binary alignment map (BAM) format using SAMtools (v1.22.1). Gene-level counts were generated with GenomicFeatures package (v1.35.11) from Bioconductor using the UCSC rn6 Ensembl GTF annotation file.

Analyses were conducted in R (v4.5.0) within RStudio (12 August 2025). Differential gene expression analysis was performed with DESeq2 with an FDR-adjusted *p*-value threshold of 0.10. Principal component analysis (PCA) was used to assess sample clustering, and the results were visualized using ggplot2 (v3.4.1). Gene set enrichment analysis (GSEA) was performed using gseGO from the clusterProfiler package to evaluate pathway-level trends independent of individual differentially expressed genes. All genes were ranked by log2 fold-change across the three exposure groups. PCA plots and pathway enrichment data are provided in Supplementary Materials, Figure S4 and Table S14, respectively.

### 2.5. Proteomics

#### 2.5.1. Protein Isolation and Proteolytic Digestion

Approximately 0.047 ± 0.004 g of lung tissue samples (*n* = 19) were lysed in TRIzol^®^ (Invitrogen, Thermo Fisher Scientific) according to the manufacturer’s instructions, supplemented with a protease and phosphatase inhibitor cocktail (100X Halt™ Protease Inhibitor Cocktail, Thermo Fisher Scientific). Protein concentrations were determined using a bicinchoninic acid assay (Pierce BCA Protein Assay Kit, Thermo Fisher Scientific). Total protein (50 µg per sample) was diluted in Tris-HCl buffer (50 mM; pH 8.0; in diethyl pyrocarbonate [DEPC]-treated water) and submitted to the Protein Facility at Iowa State University (Ames, IA, USA) for proteomic analysis.

A bovine serum albumin control (50 µg total protein; 50 mM Tris-HCl, pH 8.0; BSA) and a pooled master control (1 µg per sample) were prepared alongside each experimental sample (25 µg of protein). Samples were reduced with dithiothreitol (5 µL; 0.1 M; final concentration, 5 mM in water), and incubated at 37 °C for 30 min, followed by alkylation with iodoacetamide (1.5 µL; 1.0 M; final concentration, 15 mM in water) for 30 min at ambient temperature in the dark. Proteins were digested overnight for ~16 h at 37 °C using a trypsin/Lys-C protease mixture (Promega Corporation, Madison, WI, USA) at an enzyme-to-protein ratio of 1:25 (4 µL; *w*/*w*). Digestion was quenched with formic acid (5 µL; 1% in water; *v*/*v*), followed by centrifugation at 14,000–16,000 g for 10 min. Supernatants were desalted using C18 reversed-phase spin columns (BioPureSPN Mini HEM S18V.20, Higgins Analytical Inc., Mountain View, CA, USA) and dried by vacuum centrifugation (Savant™ SpeedVac™ Plus Concentrator, Thermo Fisher Scientific). The pooled master control and experimental samples were reconstituted in triethylammonium bicarbonate (100 µL; 100 mM; TEAB) to a final peptide concentration of 0.25 µg/µL, and peptide concentrations were quantified using Pierce™ Quantitative Colorimetric Peptide Assay (Cat No. A23275, Thermo Fisher Scientific).

#### 2.5.2. TMT Labeling and LC-MS/MS Analysis

Peptides were labeled using TMT as previously described [36]. Briefly, the pooled master control and individual samples (25 µg each) were incubated with TMT11-131C Label Reagent (0.25 mg; Cat. No. PIA34808, Thermo Fisher Scientific). Each sample was randomly assigned to one of eleven TMT tags. Samples were incubated for 1 h at ambient temperature and quenched with hydroxylamine (8 µL; 5% in 100 mM TEAB) for 15 min. Each labeled sample (1 µg) and pooled master control (1 µg) were combined into a single multiplexed sample (11 µg total), dried by vacuum centrifugation, and reconstituted in Buffer B (5%; 1 µg/µL; 80% acetonitrile, 20% double-distilled water, both containing 0.1% formic acid). We analyzed two TMT batches to accommodate all samples. The pooled master control was included in each batch and used as a reference for cross-batch normalization to minimize batch effects.

Proteomic analysis of the BSA control, sample fractions, and pooled master control sample was performed on a Q Exactive™ Hybrid Quadrupole-Orbitrap Mass Spectrometer equipped with a higher-energy collisional dissociation (HCD) fragmentation cell with a Nanospray Flex™ Ion source (Thermo Fisher Scientific). For each run, total peptide (5 µg) was loaded onto a reverse-phase C18 column (75 µm × 50 cm, double nanoViper™ PepMap™ Neo, 2 µm particle size, Cat. No. DNV7550PN, Thermo Fisher Scientific) fitted with a Nano Bore Emitter (stainless steel, 40 mm length, 1/32-inch outer diameter, Cat. No. NC0379152, Thermo Fisher Scientific).

Peptide separation was performed on an Easy-nLC 1200 System (Thermo Fisher Scientific) in gradient mode at 200 nL/min. The column was first equilibrated with Buffer A (0.1% formic acid in double-distilled water), after which peptides were eluted with Buffer B using a multi-step gradient: 0–35% Buffer B over 210 min, 35–70% over 10 min, 70–100% over 2 min, followed by a 2 min hold at 100% for column washing. Re-equilibration between injections was achieved through alternating cycles of 100% and 2% Buffer B.

MS1 acquisition was performed in positive ion mode with a resolving power of 70,000, an automatic gain control (AGC) target of 1 × 10^6^, maximum injection time of 80 ms, and an *m*/*z* scan range of 400 to 2000. For MS2, the instrument selected the 20 most abundant precursor ions per chromatographic peak, excluding trypsin autolysis products and known contaminants, for HCD fragmentation. MS2 spectra were collected using a 1.2 *m*/*z* isolation window, normalized collision energy of 32%, AGC target of 1 × 10^5^, maximum injection time of 50 ms, and a resolving power of 35,000.

#### 2.5.3. Proteome Discoverer Data Processing

Raw data were processed in Proteome Discoverer v3.0.4.388 (Thermo Fisher Scientific) for protein identification and relative quantification with the Reporter Ions Quantifier in the consensus workflow. The pooled master control sample was used for normalization across samples. MS2 spectra were searched against the *Rattus norvegicus* UniProt/SwissProt databases using the Sequest HT algorithm with full tryptic specificity, up to two missed cleavages, precursor mass tolerance of 10 ppm, and a fragment mass tolerance of 0.05 Da. Static modifications included carbamidomethylation of cysteine (+57.021 Da), while dynamic modifications included methionine oxidation (+15.995 Da), deamidation of asparagine/glutamine (+0.984 Da), and TMT11plex labeling on lysine residues and peptide N-termini (+229.163 Da). Peptide identifications were validated using MOWSE-like decoys with a maximum Δ Cn of 0.05 and strict false discovery rate (FDR) thresholds (PSM FDR ≤ 0.01; peptide FDR ≤ 0.01). High-confidence peptides (≥6 amino acids) were assembled into protein groups with a final protein-level FDR of 0.01. Quantification was based exclusively on unique peptides, with PSM intensities summed per TMT channel, normalized to the channel with the highest total intensity, and scaled to an average of 100. The resulting PSM-level report was used for downstream statistical analysis.

#### 2.5.4. Bioinformatics Analysis

Bioinformatics analyses were conducted with Amica v3.0.1 [37], following a workflow that included data filtering, normalization, imputation, and statistical evaluation. Proteins were retained if they were identified by at least two razor peptides and three MS/MS spectra, with an additional inclusion criterion of valid quantitative values in at least three samples per experimental group. Prior to statistical analysis, data were quantile normalized. Missing values were imputed using a normal distribution-based approach with a downshift of 1.8 and a width of 0.3. Protein quantification was based on TMT reporter ion-based intensity values, and differential abundance analysis was performed with the Limma R package. Volcano plots were generated to visualize changes in protein abundance between 4-OH-PCB52-exposed and sham control groups. Residual batch effects were evaluated using PCA and coefficient of variation (CV) distributions across batches. PCA of the normalized protein abundance data showed overlap between samples from the two TMT batches, with no clear batch-specific clustering (Supplementary Materials, Figure S2), while CV distributions were comparable between batches (Supplementary Materials, Figure S3), indicating that residual batch effects were minimal.

Protein–protein interaction (PPI) networks were investigated using the Search Tool for Retrieval of Interacting Genes/Proteins [STRING, v12.0; [38]]. Markov clustering (MCL) was applied to identify groups of highly associated proteins based on stochastic flow, using an inflation parameter set to 3.0 [39,40]. In network visualizations, proteins were represented as nodes, clusters were distinguished by color, and edges indicated functional associations, with line thickness corresponding to confidence scores. Only high-confidence interactions (confidence score ≥ 0.900) were retained for downstream analysis.

To assess biological context, significantly altered proteins (FDR ≤ 0.05) were annotated through UniProt and mapped to pathways using Kyoto Encyclopedia of Genes and Genomes (KEGG) identifiers. Pathway enrichment analysis was performed against the *Rattus norvegicus* KEGG reference to identify significantly enriched pathways.

### 2.6. Statistical Analysis

All statistical analyses were performed at the individual level. Body weight, in grams, was analyzed using a two-way repeated-measures ANOVA with a Šídák multiple comparisons test. Relative tissue weights (%) were calculated as the ratio of tissue weight to body weight, in grams, and analyzed using one-way ANOVA followed by Fisher’s LSD test. 4-OH-PCB52 and metabolite concentrations are reported as mean ± standard deviation (SD). For proteomic analyses, Limma was used to compare the medium 5% and high 10% 4-OH-PCB52 exposure groups with sham controls. To account for multiple testing, *p*-values were adjusted using the Benjamini–Hochberg FDR procedure. Proteins were considered differentially abundant if they met both an FDR-adjusted *p*-value threshold of ≤ 0.05 and a log_2_ fold-change (FC) of ≥ 1.0 or ≤ −1.0. For KEGG pathway enrichment analyses, significance was determined using Benjamini–Hochberg FDR correction, with enriched pathways defined as those with FDR ≤ 0.05.

## 3. Results

### 3.1. In Vivo Dosimetry of 4-OH-PCB52 Exposure

Adolescent female rats were implanted subcutaneously with polymeric implants containing increasing mass percentages of 4-OH-PCB52. No evidence of local tissue reactions or changes in implant integrity was observed. Approximately 2.1–3.5% of 4-OH-PCB52 remained in the implants after 28 days of exposure (Table 1). The resulting internal doses of 4-OH-PCB52, assuming 100% bioavailability of the released 4-OH-PCB52, were calculated as 0, 8.2, 41.8, and 87.9 mg/kg bw for the sham, low 1%, medium 5%, and high 10% dose groups, respectively.

### 3.2. Organ Weights and Tissue Morphology

**Figure 1:**
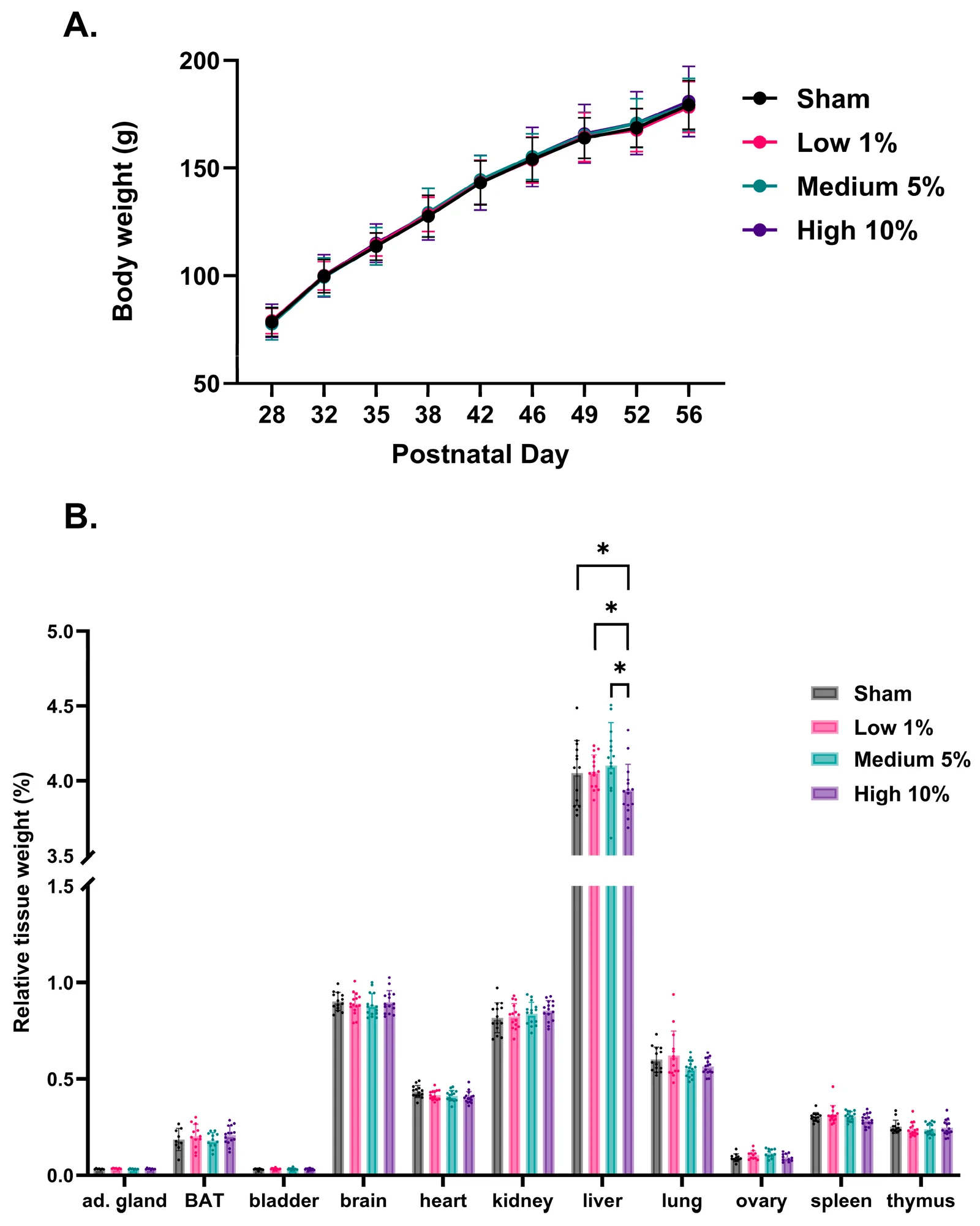
Exposure effect of 4-OH-PCB52 on body weight and relative tissue weight in female rats. (**A**) Individual data points represent body weight (g) measured from PND 28 to 56 in females exposed to sham control (black), low 1% (pink), medium 5% (teal), or high 10% (purple) exposure groups. (**B**) Bar graph illustrates relative tissue weights (%) of adrenal gland (ad. gland), brown adipose tissue (BAT), bladder, brain, heart, kidney, liver, lung, ovary, spleen, and thymus across exposure groups. Data is presented as mean ± SD (*n* = 15) for both (**A**) and (**B**). * *p* < 0.01.

Body and tissue weights were evaluated following 28-day exposure to 4-OH-PCB52 via polymeric implants. No significant differences in body weight were observed (Figure 1A). Relative liver weight was significantly reduced in the high 10% exposure group compared with sham controls (*p* < 0.01; Figure 1B) and compared with low 1% and medium 5% dose groups (*p* < 0.01 for both). No significant differences were observed in the lung and other tissue weights (Figure 1B). Histopathological examination revealed no exposure-related lung lesions (Supplementary Materials, Figure S5). Incidental bacteria were observed in alveoli from both sham and exposed animals without associated inflammation and were considered unrelated to 4-OH-PCB52 exposure.

### 3.3. Pulmonary Levels of 4-OH-PCB52 and Metabolites

To assess pulmonary accumulation of 4-OH-PCB52, concentrations of 4-OH-PCB52 and downstream metabolite 4,4′-diOH-PCB52 were measured in lung tissue from adolescent female rats following a 28-day exposure (Table 2). 4-OH-PCB52 was detected in all lung samples from the medium 5% and high 10% dose groups, with concentrations of 17 ± 4 and 25 ± 7 ng/g wet weight, respectively, but was below the limit of detection in the low 1% dose group. In contrast, 4,4′-diOH-PCB52 was not detected in lung tissue from any exposure group.

### 3.4. Transcriptomic Changes

RNA sequencing analysis was performed to evaluate transcriptional changes. PCA and hierarchical clustering did not demonstrate group separation (Supplementary Materials, Figure S4). No genes met the threshold for differential expression across any dose group relative to sham controls. However, GSEA pathway analysis highlighted 18 pathways associated with 4-OH-PCB52 exposure, including processes related to cilium assembly and movement, axoneme assembly, and microtubule bundle formation (Supplementary Materials, Table S14).

### 3.5. Proteomic Profiling

The effects of LC-PCBs, including 4-OH-PCB52, on the pulmonary proteome are poorly characterized, particularly during sensitive windows of development. Adolescence is a critical period of lung maturation that may heighten vulnerability to environmental toxicants, yet the impact of exposure to 4-OH-PCB52 during this developmental stage remains uncharacterized. To address these knowledge gaps, we conducted global proteomic profiling of lung tissue markers followed by pathway-level analyses of biological processes essential for lung function to evaluate proteomic changes associated with 4-OH-PCB52 exposure in adolescent females.

#### 3.5.1. Global Proteome Alterations

**Figure 2:**
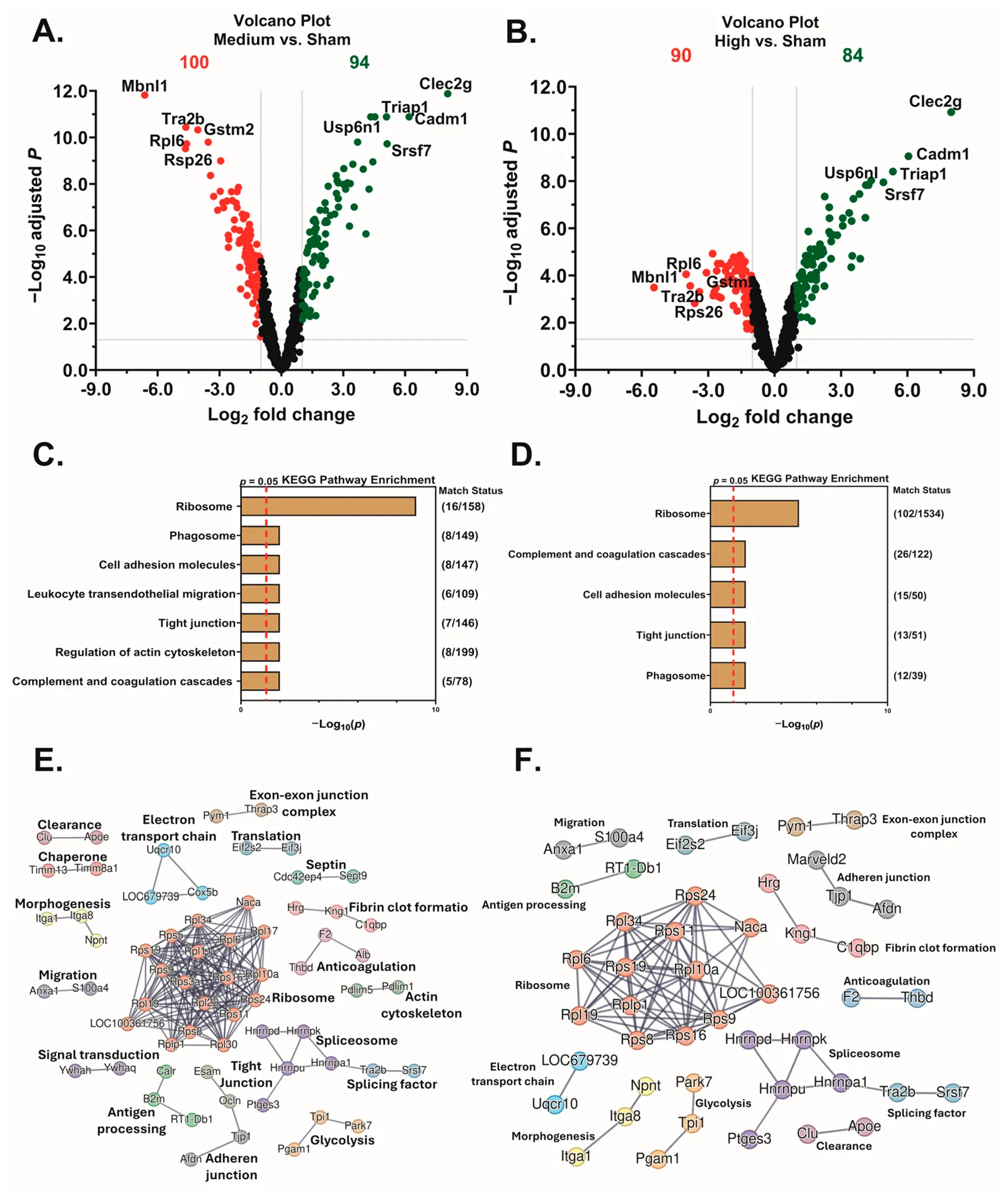
Dose-dependent changes in the pulmonary proteome altered by subcutaneous 4-OH-PCB52 exposure in adolescent female rats. Volcano plots show differentially abundant lung proteins following (**A**) medium 5% and (**B**) high 10% 4-OH-PCB52 exposure, with significance defined by FDR ≤ 0.05 and log_2_ fold-change (FC) ≥ 1.0 or ≤ −1.0. Red = decreased significant abundance, Green = increased significant abundance. No significant differences were observed between the low 1% dose and sham groups. KEGG pathway enrichments and protein-protein network clusters were performed using STRING analysis for the medium 5% (**C**,**E**) and high 10% dose exposure groups (**D**,**F**). *n* = 5 per exposure group. Multiple testing correction was performed using the Benjamini–Hochberg procedure. Cluster colors have no meaningful significance, for identification only.

PCA showed clear separation between the sham controls and 4-OH-PCB52-exposed female rats (Supplementary Materials, Figure S1). No significant proteomic differences were seen between the sham and low 1% dose groups. In contrast, relative to the sham controls, 194 lung proteins were significantly altered in the medium 5% dose group (FDR ≤ 0.05; Figure 2A and Supplementary Materials, Table S7), with 94 proteins increased and 100 decreased in abundance. Similarly, the high 10% dose group showed significant alterations in 174 proteins compared with sham (FDR ≤ 0.05; Figure 2B and Supplementary Materials, Table S9), including 84 increased and 90 decreased proteins.

Among the most reduced protein abundance in both exposure groups were muscleblind-like splicing regulator 1 (MBNL1), transformer 2 beta homolog (TRA2B), ribosomal protein S26 (RPS26), ribosomal protein L6 (RPL6), and glutathione s-transferase mu 2 (GSTM2). Likewise, the most increased proteins in both groups included C-type lectin domain family 2 member G (CLEC2G), cell adhesion molecule 1 (CADM1), TP53-regulated inhibitor of apoptosis 1 (TRIAP1), serine/arginine-rich splicing factor 7 (SRSF7), and USP6 N-terminal like protein (USP6NL).

KEGG pathway enrichment analysis of differentially abundant lung proteins (FDR ≤ 0.05) identified dose-dependent differences across multiple pulmonary pathways in both the medium 5% (Figure 2C; Supplementary Materials, Table S8) and high 10% dose (Figure 2D; Supplementary Materials, Table S10) exposure groups. Enriched pathways common to both doses included “ribosome”, “complement and coagulation cascades”, “tight junction”, “cell adhesion molecules”, and “phagosome” pathways. “Leukocyte transendothelial migration” and “regulation of actin cytoskeleton” pathways were enriched exclusively in the medium 5% dose group.

Protein–protein interaction network analysis identified 54 and 41 significantly altered nodes in the medium 5% and high 10% dose groups, respectively (Figure 2E,F). Comparative analysis of lung proteins between exposure doses further identified 172 proteins that were commonly altered across exposure groups (FDR ≤ 0.05; Supplementary Materials, Table S11).

#### 3.5.2. Gene Expression Machinery

**Figure 3:**
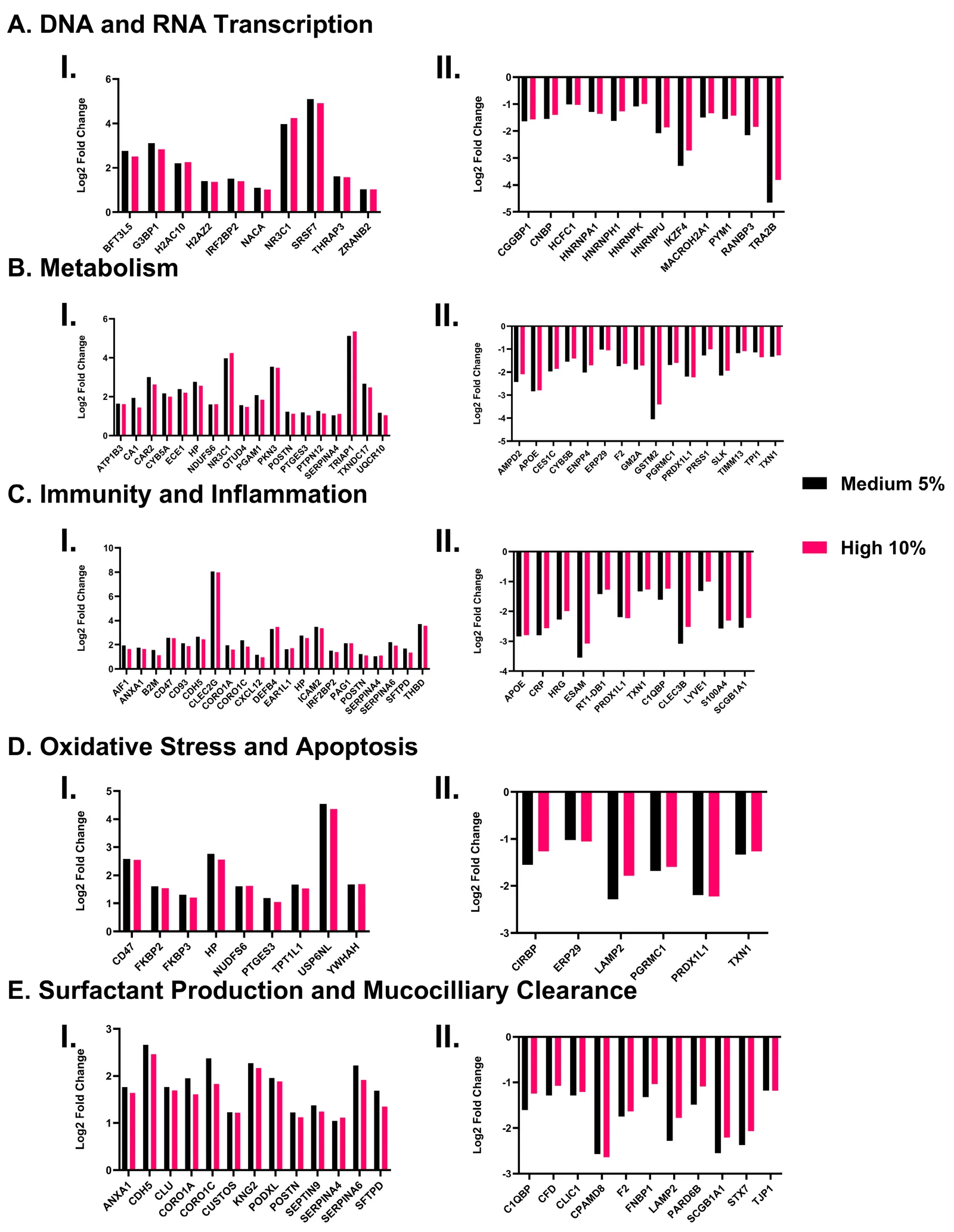
Physiological functions of differentially abundant lung proteins are illustrated by log2 fold-change. (**A**) DNA and RNA transcription, (**B**) metabolism, (**C**) immunity and inflammation, (**D**) oxidative stress and apoptosis, and (**E**) surfactant production and mucociliary clearance in the lung following 4-OH-PCB52 exposure in female adolescent rats. Panels (**I**) and (**II**) represent increased and decreased log2 fold-change, respectively, in medium 5% (*n* = 5; black) and high 10% (*n* = 5; pink) dose exposure groups relative to sham control. No significant differences were observed in the low 1% dose relative to sham control. Differential abundance was defined by an FDR ≤ 0.05; log_2_ fold-change (FC) ≥ 1.0 or ≤ −1.0.

Using global proteomics, we found 22 proteins altered by 4-OH-PCB52 exposure related to chromatin organization, transcriptional regulation, RNA processing, and mRNA translation. Compared to sham control rats, medium 5% and high 10% dose increased abundance (log2 fold-change ≥ 1.0; FDR ≤ 0.05; Figure 3(AI)) of histone variants and chromatin-associated regulators (H2AC10 and H2AZ2), transcriptional and post-transcriptional regulators (BTF3L5, IRF2BP2, SRSF7, THRAP3, and ZRANB3), RNA binding and translational regulation (G3BP1 and NACA), and nuclear receptors (NR2C1). Decreased abundance (log2 fold-change ≤ −1.0; FDR ≤ 0.05; Figure 3(AII)) of proteins involved processes related to chromatin-associated and transcriptional regulators (CGGBP1, CNBP, HCFC1, and MACROH2A1), RNA-binding and splicing-related proteins (HNRNPA1, HNRNPH1, HNRNPK, HNRNPU, and TRA2B), mRNA processing and export (PYM1 and RANBP3), and transcription factors (IKZF4).

#### 3.5.3. Metabolic and Xenobiotic Pathways

Proteomic analysis revealed significant alterations to 34 lung proteins in both medium 5% and high 10% dose 4-OH-PCB52 samples involved in cellular metabolism, mitochondrial function, and redox homeostasis. Proteins with increased abundance (log2 fold-change ≥ 1.0; FDR ≤ 0.05; Figure 3(BI)) were enriched for markers of mitochondrial oxidative phosphorylation (NDUFS6 and UQCR10), redox-related proteins (CYB5A and TXNDC17), acid–base regulation (CA1 and CA2), glycolytic pathway proteins (PGAM1), metabolic regulation and signaling proteins (NR3C1, PKN3, PTGES3 and PTPN12), and extracellular or transport-associated proteins (HP, POSTN, and SERPINA4). In contrast, proteins with decreased abundance (log2 fold-change ≤ −1.0; FDR ≤ 0.05; Figure 3(BII)) were associated with glycolysis and nucleotide metabolism (AMPD2 and TPI1), lipid transport and biotransformation (APOE and CES1C), redox regulation (CYB5B, GSTM2, PRDX1L1, and TXN1), mitochondrial and endoplasmic reticulum function (TIMM13 and ERP29), and proteolytic activity and metabolic regulation (ENPP4, F2, GM2A, PRSS1, PGRMC1, SLK).

#### 3.5.4. Immune and Inflammatory Responses

Compared to sham controls, rats exposed to medium 5% and high 10% doses of 4-OH-PCB52 exhibited altered expression of 33 lung proteins involved in immunity and inflammation. Proteins with elevated abundance (log2 fold-change ≥ 1.0; FDR ≤ 0.05; Figure 3(CI)) included cytokines/chemokines (CXCL12 and DEFB4), macrophage/microglia activation marker (AIF1), leukocyte adhesion/migration/endothelial activation (ICAM2), macrophage/monocyte/neutrophil-associated genes (CORO1A and CORO1C), interferon-related immune regulation (IRF2BP2), immune-related (ANXA1, B2M, CD47, CD93, CLEC2G, and EAR1L1), vascular and endothelial function (CDH5 and THBD), extracellular matrix and secreted proteins (POSTN, SERPINA4, SERPINA6 and HP), and surfactant proteins (SFTPD). Proteins with reduced abundance (log2 fold-change ≤ −1.0; FDR ≤ 0.05; Figure 3(CII)) included lipid transport and inflammatory markers (APOE, CRP, and HRG), endothelial and lymphatic structures (ESAM and LYVE1), immune recognition and redox regulation (C1QBP, PRDX1L1, RT1-DB1, TXN), and extracellular and epithelial-associated proteins (CLEC3B, S100A4, and SCGB1A1).

#### 3.5.5. Oxidative Stress and Apoptosis

Altered abundance of 15 lung proteins related to the cellular stress response was observed in female rats exposed to 4-OH-PCB52 at both medium 5% and high 10% doses. Heightened abundance (log2 fold-change ≥ 1.0; FDR ≤ 0.05; Figure 3(DI)) included extrinsic apoptosis/immune-mediated cell death (CD47), chaperone-associated proteins (FKBP2, FKBP3, PTGES3, TPT1L1, USP6NL, and YWHAH) and mitochondrial respiration (NDUFS6). In contrast, proteins with reduced abundance (log2 fold-change ≤ −1.0; FDR ≤ 0.05; Figure 3(DII)) included RNA binding and endoplasmic reticulum stress-induced apoptosis (CIRBP and ERP29), lysosomal/autophagy–apoptosis crosstalk (LAMP2), and intrinsic apoptosis/mitochondrial pathway markers (PRDX1L1 and TXN1).

#### 3.5.6. Surfactant and Mucociliary Function

Changes in the abundance of 24 lung proteins involved in surfactant-associated processes and secreted components were observed at both medium 5% and high 10% doses. In addition to surfactant-associated protein (SFTPD), discussed above, secreted chaperone (CLU) was increased in 4-OH-PCB52-exposed females. Additionally, airway epithelial and endothelial structure and polarity markers (CDH5, PODXL, and SEPTIN9), cytoskeletal and actin-associated proteins (CORO1A and CORO1C), and extracellular matrix and circulating or secreted proteins (ANXA1, CUSTOS, KNG2, POSTN, SERPINA4, SERPINA6) were elevated following 4-OH-PCB52 exposure (log2 fold-change ≥ 1.0; FDR ≤ 0.05; Figure 3(EI)). Reduced abundance (log2 fold-change ≤ −1.0; FDR ≤ 0.05; Figure 3(EII)) included markers associated with epithelial polarity, vesicular trafficking, and secretory function (FNBP1, LAMP2, PARD6B, STX7, and TJP1), airway epithelial secretion (SCGB1A1), and airway surface hydration and mucous homeostasis (CFD, CLIC1, CPAMD8, and F2).

## 4. Discussion

Polychlorinated biphenyls (PCBs) were widely used in industrial applications, leading to persistent environmental contamination and continued human exposure through diet, inhalation, and dermal contact [5]. PCB52 is a lower-chlorinated PCB congener. In humans, biotransformation of PCB52 involves oxidation of the parent compound by the phase I enzyme CYP2A6 to 4-OH-PCB52 [14]. This metabolite has demonstrated cytotoxicity in a human preadipocyte cell line [13], potentially altering biological activity at the cellular level, a finding that warrants in vivo investigation. In rats, alveolarization continues through adolescence (PND 14 to 60), paralleling key stages of postnatal lung development in humans [41,42]. Thus, the exposure period used in this study (PND 28 to 56) corresponds to a critical window of lung maturation. Using a polymeric implant to achieve sustained systemic exposure to the human-relevant PCB metabolite 4-OH-PCB52 over 28 days, we observed significant alterations in the pulmonary proteome of adolescent female rats.

To place these biological observations into the context of exposure relevance, it is important to consider the internal doses achieved and the disposition of 4-OH-PCB52 during this study. The corresponding doses were based on estimated lifetime human PCB52 exposures via inhalation and oral routes, accounting for PCB52 biotransformation to 4-OH-PCB52, absorption efficiency, bioavailability, and body weight assumptions [30]. Over 28 days, polymeric implants released 96.5 to 97.9% of the loaded compound, yielding estimated internal doses of 8.2, 41.8, and 87.9 mg/kg bw for the low-, medium-, and high-dose groups, respectively. These doses are comparable to those observed in our earlier study with male rats [30,31], and the minimal residual compound in the implant at the end of the study supports a reproducible, sustained systemic release of 4-OH-PCB52 throughout the exposure period. 4-OH-PCB52 was detected in lung tissue from the medium- and high-dose groups at concentrations of 17 ± 4 ng/g and 25 ± 7 ng/g wet weight, respectively, but was not detected in the low-dose group. Despite detectable pulmonary concentrations of the parent metabolite, the dihydroxylated metabolite 4,4′-diOH-PCB52 was not detected, in contrast to findings in similarly exposed male rats [30]. These differences between studies may be due to sex-specific differences in PCB metabolism or to methodological variability, such as differences in analytical sensitivity across studies.

Body weight did not differ across groups from PND 28 to 56, indicating that 4-OH-PCB52 exposure did not cause overt systemic toxicity or impaired growth. Relative liver weight was significantly reduced in the high exposure group compared to the other groups, a result similar to the male rat study [30]. In contrast, PCB mixtures generally increase liver weight in rats [43,44], an effect commonly attributed to persistent AhR activation by higher-chlorinated congeners that resist metabolism and accumulate in hepatic tissue [45]. Thus, the hepatic effects of 4-OH-PCB52 at higher exposure levels appear to differ from those of PCB mixtures. Transcriptomic alterations were observed in the liver of adolescent female rats following inhalation exposure to PCB52 [46], indicating that some of the hepatic transcriptomic changes associated with PCB52 inhalation may be mediated by its metabolites, such as 4-OH-PCB52. Although hepatic endpoints were not a primary focus of the present study, the liver and lung are linked through shared inflammatory and immune signaling pathways, such as macrophage activation and cytokine-mediated signaling, with responses in one organ influencing the other through circulatory mediators [47,48]. Therefore, the reduction in liver weight may reflect metabolic or immunological adaptations to 4-OH-PCB52 exposure, although further studies evaluating hepatic endpoints are needed to determine the underlying mechanisms.

Although evidence of systemic effects was observed in the liver, we next assessed whether comparable responses occurred in the lung. Relative lung weight did not differ significantly among exposure groups, and histopathological evaluation revealed no exposure-related alterations in lung morphology, similar to the earlier study in male rats [30]. This contrasts with a previous 28-day inhalation study, which reported increased relative lung weight in PCB52-exposed female rats [9]. This discrepancy may reflect differences in exposure route, as inhalation delivers the parent compound directly to the lung, whereas the subcutaneous implants provide systemic exposure to the metabolite at lower lung concentrations. These results suggest that, under the conditions of the present study, 4-OH-PCB52 exposure did not induce overt structural changes in the lung despite evidence of systemic effects reflected by reduced liver weight.

To determine whether molecular alterations were present despite the absence of overt morphological changes, transcriptomic and proteomic analyses were performed. GSEA of the transcriptomics data identified cilium-related pathways significantly altered by 4-OH-PCB52 exposure in the medium- and high-dose groups; however, individual genes were not differentially expressed at an FDR threshold of 0.10. These results may indicate transcriptional changes below the threshold of gene-level significance [49]. In contrast, as discussed below, 4-OH-PCB52 caused significant alterations in the lung proteome. The mismatch between transcriptomic and proteomic responses may reflect the multiple regulatory processes that occur between transcription and protein synthesis. Several non-mutually exclusive factors may contribute to this discordance, including post-transcriptional mechanisms (e.g., mRNA stability, translational regulation, and microRNA activity), and differences in transcript versus protein turnover kinetics, as mRNA changes are typically transient while proteins persist for longer. Thus, transcriptomic and proteomic datasets may capture different stages of the biological response, particularly at a single time point [50,51]. Lastly, the sample size may have reduced the statistical power of the RNA-sequencing analysis, potentially contributing to the observed differences between the transcriptomic and proteomic datasets. However, the absence of significantly differentially expressed genes does not exclude the possibility that transcriptional regulation occurred in response to 4-OH-PCB52 exposure.

Given these limitations and the absence of significant gene-level changes, proteomics provided an important complementary approach for evaluating biological response to 4-OH-PCB52 exposure. Consistent with this interpretation, principal component analysis showed overlap between sham and low 4-OH-PCB52-exposed animals, indicating no significant proteomic changes at this exposure level. This observation likely reflects limited biological effects at the statistical threshold and is consistent with the lack of detectable 4-OH-PCB52 in lung tissue, as concentrations were below the analytical limit of detection. In contrast, the medium- and high-dose groups exhibited similar numbers of differentially expressed proteins, despite a 2-fold difference in administered dose, consistent with either a threshold effect in the proteomic response or release kinetics of 4-OH-PCB52 from the polymeric implant over the 28-day study period that yielded more similar effective exposures than expected from the nominal doses.

KEGG pathway analysis of the proteomics data revealed enrichment of ribosome, complement and coagulation cascade, tight junction, cell adhesion molecule, and phagosome pathways in the medium- and high-exposure groups. Enrichment of pathways associated with tight junctions, cell adhesion molecules, and phagosomes may be due to exposure-associated proteomic changes in biological processes involved in epithelial barrier function and immunity. Notably, enrichment of leukocyte transendothelial migration and actin cytoskeleton regulation pathways was unique to the medium-dose group, suggesting at this dose there may be early immune cell recruitment or cytoskeletal remodeling. These pathway-level associations are consistent patterns reported following exposure to other pulmonary irritants, including silica, zinc chloride smoke, and engineered nanomaterials [52,53,54], extending these observations to a hydroxylated PCB metabolite. To better understand the biological significance of these pathway enrichments, we next examined individual proteins that contributed to the observed exposure-associated responses.

The most notable evidence at the individual protein level was decreased club cell secretory protein (SCGB1A1, also known as CC10 or CC16) and increased surfactant protein D (SFTPD) in the lungs of 4-OH-PCB52-exposed rats. SCGB1A1 was originally identified as a lung PCB-binding protein, with a PCB52 methylsulfonyl metabolite serving as a high-affinity ligand [55]. Reduced levels of its human ortholog have been reported in smokers [56], providing a precedent for the decrease observed here following exposure to a PCB metabolite. Reduced CC16 levels in childhood have been associated with persistent deficits in lung function, while CC16-deficient mice develop spontaneous pulmonary inflammation and airway remodeling [57,58]. Therefore, the reduction in SCGB1A1 observed here may reflect club cell stress, impaired epithelial stability, or altered secretion in response to 4-OH-PCB52 exposure. In contrast, SFTPD abundance was increased in exposed lungs. SFTPD, produced by alveolar type II cells and club cells, plays key roles in surfactant equilibrium and innate immune defense [59]. Elevated SFTPD is a well-established marker of pulmonary inflammation and alveolar injury [60,61]; thus, the SCGB1A1-down/SFTPD-up signature may reflect disruption of airway homeostasis and mucosal defense mechanisms involved in lung injury [58,62]. It should be noted that the subcutaneous implant model and resulting internal doses used in this study do not directly replicate a human environmental exposure scenario. Nonetheless, 4-OH-PCB52 is a physiologically relevant human PCB metabolite, and extrapolation of these molecular findings to human respiratory disease should be made with appropriate caution.

Supportive of the evidence for altered epithelial homeostasis, several proteins involved in maintaining epithelial barrier structure and function were also affected. Reduced abundance of the barrier-associated proteins TJP1 and PARD6B, along with enrichment of the tight junction pathway, indicates potential changes to epithelial integrity and polarity [63,64,65]. Previous studies show PCB disruption of barrier integrity in intestinal epithelial cells through NADPH oxidase-driven oxidative stress, resulting in reduced occludin and TJP1 expression [66]. CDH5 and PODXL are involved in endothelial junction integrity and leukocyte transmigration, respectively; reduced abundance of both proteins, alongside enrichment of the leukocyte transendothelial migration pathway in the medium-dose group, suggests a possible molecular basis for altered vascular permeability and immune cell trafficking that was not functionally tested.

Proteins associated with immune and inflammatory processes exhibited altered abundance, including increased CXCL12, AIF1, ANXA1, and CORO1A/1C, markers of immune cell recruitment and macrophage activation [67,68,69], alongside reduced complement regulatory proteins, such as APOE, CRP, HRG, ESAM, LYVE1, C1QBP, and RT1-DB1. The increased abundance of immune proteins is similar to prior reports that PCB126 impairs neutrophil GPCR-driven oxidative burst following pulmonary accumulation [70], suggesting that PCBs can modulate innate immune function in the lung through diverse mechanisms. Furthermore, ANXA1 increased abundance may represent a compensatory anti-inflammatory response [71]. These findings, in agreement with the enrichment of the complement and coagulation cascade pathway, indicate potential activation of innate immune defense mechanisms, although the mixed directionality of individual proteins supports a complex rather than uniform response to 4-OH-PCB52 exposure.

In addition to immune-related alterations, the proteomic profile also suggests disruption of cellular redox homeostasis. This finding is akin to the known reactivity of 4-OH-PCB52 and its quinone metabolites, which are electrophilic and can form adducts with glutathione and proteins [15]. Decreased abundance of redox defense proteins GSTM2, TXN1, and peroxiredoxins may demonstrate sustained oxidative stress and impaired antioxidant defense in the lung [72]. Unlike the induction of antioxidant enzymes observed following dioxin-like PCB exposure in the liver [73,74], this response may reflect the weak AhR activity of 4-OH-PCB52 and tissue-specific regulation of Phase II detoxification in the lung [75].

Because electrophilic PCB quinone metabolites can also form adducts with DNA and histones, we next examined proteins involved in RNA processing and chromatin regulation. Several changes to heterogeneous nuclear ribonucleoproteins (HNRNPA1, HNRNPH1, HNRNPK, HNRNPU) and histone variants (H2AC10, H2AZ2, macroH2A1) were observed. Since hnRNPs regulate mRNA splicing, stability, and translation [76], their depletion may contribute to the transcriptome-proteome changes observed in this study by altering RNA processing without affecting steady-state mRNA levels [77,78]. Thus, reduced macroH2A1 abundance may alter chromatin organization in response to the genotoxic potential of PCB quinones to form adducts with DNA and histones [79].

Given the close interconnection between oxidative stress and mitochondrial dysfunction, we next examined proteins associated with mitochondrial function. Increased abundance of the electron transport chain components NDUFS6 (Complex I) and UQCR10 (Complex III) may reflect a mitochondrial adaptation to cellular stress. Because 4-OH-PCB52 has been shown to reduce mitochondrial membrane potential and impair mitochondrial function [80], the observed increase in OXPHOS-related proteins may reflect a compensatory response to mitochondrial stress and impaired bioenergetic function [81]. Additionally, reduced abundance of TIMM13 and CYB5B, alongside elevated CYB5A, further supports alterations in mitochondrial protein import and electron transfer processes [82,83]. Consistent with previously reported PCB52-induced mitochondrial gene expression changes in the brain of adolescent male rats [10], mitochondrial dysfunction may be a response to PCB exposure across tissues.

While our findings support the conclusion that 4-OH-PCB52 affects multiple biological processes in the lung, several study limitations should be considered. The study evaluated only a 28-day exposure duration and had a modest sample size, particularly for the transcriptomic analyses. Internal dose estimates were calculated assuming complete bioavailability of the 4-OH-PCB52 released from the implants; this assumption may overestimate actual systemic exposure, as local retention and metabolism could reduce the fraction of the released compound that reaches systemic circulation. Proteomic findings were based on LC-MS/MS alone, without orthogonal validation (e.g., Western blot or immunohistochemistry) or functional assays to confirm individual protein changes or their physiological consequences; the affected proteins and pathways identified here should therefore be considered discovery-level candidates requiring confirmation in future studies. Molecular and histopathological changes were assessed, but physiological respiratory endpoints (e.g., respiratory rate and breathing parameters) were not evaluated. Similarly, sex-specific differences in lung responses could not be assessed, despite well-documented sex-related changes in hormone signaling, xenobiotic metabolism, and immune function [32]. In addition, the proteomic analysis lacked cell-type-specific resolution, potentially masking responses in individual lung cell populations.

## 5. Conclusions

This study provides a global proteomic characterization of pulmonary responses to subacute exposure to 4-OH-PCB52 in adolescent female rats and identifies alterations in proteins associated with respiratory homeostasis. These findings highlight the biological activity of hydroxylated PCB metabolites and identify candidate biomarkers and pathways relevant to both experimental models and human populations. Further studies should examine sex-specific and temporal responses, incorporate cell-type-specific analyses, and determine whether these molecular alterations translate into functional and adverse respiratory outcomes.

## Figures and Tables

**Table 1:** Estimated dosimetry of 4-OH-PCB52 following 28-day polymeric implant exposure in adolescent female rats.

4-OH-PCB52 Exposure Metrics	Exposure Groups ^1^			
	Sham	Low 1%	Medium 5%	High 10%
Total 4-OH-PCB52 load in the implant (mg) ^2^	0	1.1 ± 0.1	5.6 ± 0.1	11.8 ± 0.2
4-OH-PCB52 remaining in the implant (mg) ^2^	0	0.02 ± 0.01	0.20 ± 0.05	0.4 ± 0.1
4-OH-PCB52 remaining in the implant (%) ^2^	0	2 ± 1	3.5 ± 0.9	3.5 ± 0.9
Total dose (mg/kg) ^2,3^	0	8.2 ± 0.4	42 ± 3	88 ± 8

^1^ *n* = 15 per exposure group; ^2^ mean ± SD; ^3^ body weight represents the time-weighted average over 28 days.

**Table 2:** Levels (ng/g wet weight) and detection frequency (%) of 4-OH-PCB52 and 4,4′-diOH-PCB52 determined by GC-MS/MS in the lung of 4-OH-PCB52 polymeric implant exposure groups ^1^.

Analyte Class	Specific Analyte	Analytical Method ^2^	Limits of Detection ^3^	4-OH-PCB52 Levels ^4^ (ng/g Wet Weight)			Number of Detects/Number of Samples (Detection Frequency, %)		
				Low 1%	Medium 5%	High 10%	Low 1%	Medium 5%	High 10%
OH-PCB	4-OH-PCB52	GC-MS/MS	8.7	<LOD	17 ± 4	25 ± 7	0/4 (0)	5/5 (100)	5/5 (100)
diOH-PCB	4,4′-diOH-PCB52		2.7	<LOD	<LOD	<LOD	0/4 (0)	0/5 (0)	0/5 (0)

^1^ Low 1%, *n* = 4; medium 5%, *n* = 5; high 10%, *n* = 5; ^2^ GC-MS/MS = gas chromatography–mass spectrometry/mass spectrometry; ^3^ LOD, limit of detection (ng/g), was calculated based on matrix blanks for each tissue using the formula LOD = mean_blank_ + t_0.01,*n*−1_ * SD_blank_, where mean_blank_ is the mean of blank measures, t_0.01,*n*−1_ is Student’s t-value for *n* − 1 degrees of freedom at the 99% confidence level, and SD_blank_ is the standard deviation of the blank measures; *n* = 5; S/N = signal to noise ratio; ^4^ Mean ± SD.

## Data Availability

The original data presented in the study are openly available in the Iowa Research Online repository, https://doi.org/10.25820/data.008280. Transcriptomic data have been deposited in the Gene Expression Omnibus (GEO) under accession number GSE339138. Proteomics data have been deposited in the ProteomeXchange Consortium (PXD081489) via the MassIVE repository (MSV000102563) under the DOI 10.25345/C50C4T036 (https://doi.org/doi:10.25345/C50C4T036).

## Supplementary Materials

The following supporting information can be downloaded at: https://www.mdpi.com/article/10.3390/jox16060189/s1. Figure S1: Principal component analysis (PCA) plot of lung proteomic samples; Figure S2: Principal component analysis (PCA) of normalized proteomic data across TMT batches; Figure S3: Coefficient of variation (CV) distributions across TMT batches; Figure S4: Principal component analysis (PCA) of lung transcriptomic profiles; Figure S5: Representative images for the hematoxylin and eosin (H&E) staining of the lung histopathology in female PND56 rats; Table S1: Abbreviations and unique identifiers of the test compounds and analytical standards used in this study; Table S2: Precursor ions, product ions, and collision energies for each analyte included in the GC-MS/MS analysis; Table S3: Recoveries (%) of the ongoing precision and recovery standard spiked into matrices from adolescent female rats in the GC-MS/MS Analysis; Table S4: Surrogate standard recoveries (%) from all tissues in the GC-MS/MS analysis; Table S5: Method detection limits (ng) and limits of detection for each tissue type (ng/g) for the GC quantifications of 4-OH-PCB52 and 4,4′-diOH-PCB52 metabolite; Table S6: Levels (ng/g) of 4-OH-PCB52 and 4,4′-diOH-PCB52 in lung tissue for each exposure group; Table S7: Lung proteins significantly altered by medium 5% 4-OH-PCB52 exposure; Table S8: KEGG enrichment pathways associated with medium 5% 4-OH-PCB52 exposure; Table S9: Lung proteins significantly altered by high 10% 4-OH-PCB52 exposure; Table S10: KEGG enrichment pathways associated with high 10% 4-OH-PCB52 exposure; Table S11: Common lung proteins altered between medium 5% and high 10% exposure of 4-OH-PCB52; Table S12: Principal component analysis loadings for proteomics; Table S13: Summary of RNA-sequencing read counts and quality control metrics for lung transcriptome libraries; Table S14: Gene enrichment pathways associated with lung transcriptomics.

## Abbreviations

The following abbreviations are used in this manuscript:

AGC	Automatic gain control
ANOVA	Analysis of variance
BAM	Binary alignment map
BSA	Bovine serum albumin
DEP	Differentially expressed proteins
FDR	False discovery rate
GC-MS/MS	Gas chromatography–tandem mass spectrometry
GSEA	Gene set enrichment analysis
HCD	Higher-energy collisional dissociation
HCl	Hydrochloric acid
KEGG	Kyoto Encyclopedia of Genes and Genomes
LC-MS/MS	Liquid chromatography–tandem mass spectrometry
LC-PCBs	Lower-chlorinated polychlorinated biphenyls
LOD	Limit of detection
LSD	Least significant difference
MCL	Markov clustering
MDL	Method detection limit
MRM	Multiple reaction monitoring
MS1	First-stage mass spectrometry
MS2	Second-stage or tandem mass spectrometry
OH-PCBs	Hydroxylated polychlorinated biphenyls
OPR	Ongoing precision and recovery
PCA	Principal component analysis
PCBs	Polychlorinated biphenyls
PND	Postnatal day
ppm	Parts per million
PSM	Peptide-spectrum matches
QA/QC	Quality assurance/quality control
RIN	RNA integrity number
SAM	Sequence Alignment/Map
SD	Standard deviation
TMT	Tandem mass tags
