# Supplementary material for: Pulmonary Proteomic Responses to Subacute 2,2′,5,5′-Tetrachlorobiphenyl-4-ol Exposure via a Subcutaneous Implant in Adolescent Female Rats
Source: J Xenobiot. 2026 Oct 6;16(6):189. doi: 10.3390/jox16060189 (PMC13706538; doi:10.3390/jox16060189)
Supplement: Supplementary file 1 [file jox-16-00189-s001.zip › jox-4440610-supplementary.pdf]

Pulmonary Proteomic Responses to Subacute 2,2',5,5'-Tetrachlorobiphenyl-4-ol Exposure via a Subcutaneous Implant in Adolescent Female Rats

Crystal M. Roach, Hui Wang, Nate R. Koester, Joel D. Nott, Xueshu Li, and Hans-Joachim Lehmler[1]

[1]**Corresponding Author:**
Dr. Hans-Joachim Lehmler
The University of Iowa
Department of Occupational and Environmental Health
College of Public Health
University of Iowa
145 N. Riverside Drive
Iowa City, IA 52242
Phone: +1-319-335-4981
Fax: +1-319-384-4123
Email: hans-joachim-lehmler@uiowa.edu

Table of Contents

Table S1. Abbreviations and unique identifiers of the test compounds and analytical standards used in this study. 3

Table S2. Precursor ions, product ions, and collision energies for each analyte included in the GC-MS/MS analysis. 5

Table S3. Recoveries (%) of the ongoing precision and recovery standard spiked into matrices from adolescent female rats in the GC-MS/MS analysis. Data are reported as mean ± standard deviation. 6

Table S4. Surrogate standard recoveries (%) from all tissues in the GC-MS/MS analysis. Data are reported as mean ± standard deviation. Range is reported as minimum, maximum values. 7

Table S5. Method detection limits (ng) and limits of detection for each tissue type (ng/g) for the GC quantifications of 4-OH-PCB52 and 4,4′-diOH-PCB52 metabolites. 8

Table S6. Levels (ng/g) of 4-OH-PCB52 and 4,4′-diOH-PCB52 in lung tissue for each exposure group[1]. Values are presented as the mean ± standard deviation. 9

Table S7. Lung proteins significantly altered by medium 5% 4-OH-PCB52 exposure. 10

Table S8. KEGG enrichment pathways associated with medium 5% 4-OH-PCB52 exposure. 19

Table S9. Lung proteins significantly altered by high 10% 4-OH-PCB52 exposure. 20

Table S10. KEGG enrichment pathways associated with high 10% 4-OH-PCB52 exposure. 28

Table S11. Common lung proteins altered between medium 5% and high 10% exposure of 4-OH-PCB52. 29

Table S12. Principal component analysis loadings for proteomics. 43

Table S13. Summary of RNA-sequencing read counts and quality control metrics for lung transcriptome libraries. 44

Table S14. Gene enrichment pathways associated with lung transcriptomics. 46

Figure S1. Principal component analysis (PCA) plot of lung proteomic samples. 47

Figure S2. Principal component analysis (PCA) of normalized proteomic data across TMT batches. 48

Figure S3. Coefficient of variation (CV) distributions across TMT batches. 49

Figure S4. Principal component analysis (PCA) of lung transcriptomic profiles. 50

Figure S5. Representative images for the hematoxylin and eosin (H&E) staining of the lung histopathology in female PND 56 rats 51

References .... **Error! Bookmark not defined.**

Table S1. Abbreviations and unique identifiers of the test compounds and analytical standards used in this study.

Abbreviation	IUPAC name	Formula	Isomeric SMILES	InChl	InChlKey	CAS Registry Number	Cas Registry URL	Pub Chem CID	Pub Chem Link	DTX SID	Comptox Link
4-OH-PCB25	4-hydroxy-2,4',5-trichlorobiphenyl-4-ol	C12H7Cl3O	C1=CC(=C(C=C1Cl)O)C2=CC(=C(C=C2Cl)Cl)Cl	InChI=1S/C12H7Cl3O/c13-8-3-1-7(2-4-8)12-10(15)5-6-11(16)9(12)14/h1-6,16H	QZQWZVZJXQFZFX-UHFFFAOYSA-N	68194-07-0	NA	NA	NA	NA	NA
PCB30	2,4,6-trichlorobiphenyl	C12H7Cl3	C1=CC=C(C=C1)C2=C(C=C(C=C2Cl)Cl)Cl	InChI=1S/C12H7Cl3/c13-9-6-10(14)12(11(15)7-9)8-4-2-1-3-5-8/h1-7H	MTLMVEWEYZFYTH-UHFFFAOYSA-N	35693-92-6	https://commonchemistry.cas.org/detail?cas_rn=35693-92-6	37247	https://pubchem.ncbi.nlm.nih.gov/compound/2_4_6-trichlorobiphenyl	DTXSID7073482	https://comptox.epa.gov/dashboard/chemical/details/DTXSID7073482
4-OH-PCB52	2,2′,5,5′-tetrachlorobiphenyl-4-ol	C12H6Cl4O	C1=CC(=C(C=C1Cl)C2=CC(=C(C=C2	InChI=1S/C12H6Cl4O/c13-6-1-2-	ZKDSNFDCQYBBIUUH	51274- 68-1	https://commonchemistry.c	39971	https://pubchem.ncbi.nlm.	DTXSID10199272	https://comptox.epa.gov/dashbo

Abbreviation	IUPAC name	Formula	Isomeric SMILES	InChl	InChlKey	CAS Registry Number	Cas Registry URL	Pub Chem CID	Pub Chem Link	DTX SID	Comptox Link
			Cl)O)Cl)Cl	9(14)7(3-6)8-4-11(16)12(17)5-10(8)15/h1-5,17H	FFFAOYSA -N		as.org/detail?cas_rn=51274-68-1		nih.gov/compound/39971		ard/DTXSID10199272
4,4′-diOMe-PCB52	4,4′-dimethoxy-2,2′,5,5′-tetrachlorobiphenyl	C12H6Cl4O2	C1=C(C(=CC(=C1Cl)O)Cl)C2=CC(=C(C=C2Cl)O)Cl	InChI=1S/C12H6Cl4O2/c13-7-3-11(17)9(15)1-5(7)6-2-10(16)12(18)4-8(6)14/h1-4,17-18H	RRTQNLFZFMNNPR-UHFFFAOYSA-N	NA	NA	85746645	https://pubchem.ncbi.nlm.nih.gov/compound/85746645	NA	NA
PCB204	2,2',3,4,4',5,6,6'-octachlorobiphenyl	C12H2Cl8	C1=C(C=C(C(=C1Cl)C2=C(C(=C(C(=C2Cl)Cl)l)Cl)Cl)Cl)Cl	InChI=1S/C12H2Cl8/c13-3-1-4(14)6(5(15)2-3)7-8(16)10(18)12(20)11(19)9(7)17/h1-2H	JDZUWXRNKHXZFE-UHFFFAOYSA-N	74472-52-9	https://commonchemistry.cas.org/detail?cas_rn=74472-52-9	91721	https://pubchem.ncbi.nlm.nih.gov/compound/91721	DTXSID7074240	https://comptox.epa.gov/dashboard/chemical/details/DTXSID7074240

Table S2. Precursor ions, product ions, and collision energies for each analyte included in the GC-MS/MS analysis.

Analyte	Precursor ion (m/z)	Product ion (m/z)	Collision energy (eV)
PCB30	258.0	186.0	25
PCB204	429.7	359.8	35
4-OH-PCB25	286.0	243.0	25
4-OH-PCB52	321.9	278.9	20
4,4′-diOMe-PCB52[1]	352.0	337.0	20

[1]methylated standard for 4,4′-diOH-PCB52

Table S3. Recoveries (%) of the ongoing precision and recovery standard spiked into matrices from adolescent female rats in the GC-MS/MS analysis. Data are reported as mean ± standard deviation.

**Analyte[1]**	**Lung**
4-OH-PCB52	92 ± 9
4,4′-diOH-PCB52	63 ± 11

[1]4-OH-PCB52, $n = 10$; 4,4′-diOH-PCB52, $n = 8$

Table S4. Surrogate standard recoveries (%) from all tissues in the GC-MS/MS analysis. Data are reported as mean ± standard deviation. Range is reported as minimum, maximum values.

**Recovery standard (4-OH-PCB25)**	**Lung (*n* = 40)**
Mean ± SD	85 ± 8
Range	59, 98

Table S5. Method detection limits (ng) and limits of detection for each tissue type (ng/g) for the GC quantifications of 4-OH-PCB52 and 4,4′-diOH-PCB52 metabolites.

**Detection limit**	**4-OH-PCB52**	**4,4′-diOH-PCB52**
MDL[1],[ng]	0.09	0.31
LOD[2], [ng/g]	8.7	2.7

[1]MDL, method detection limit, was calculated based on method blanks; 4-OH-PCB52 n = 10; 4,4′-diOH-PCB52, n = 8

[2]LOD, limit of detection, was calculated based on matrix blanks for each tissue using the formula LOD = $mean_{blank} + t_{0.01, n-1} * SD_{blank}$, where $mean_{blank}$ is the mean of blank measures, $t_{0.01, n-1}$ is Student's t-value for n – 1 degrees of freedom at the 99% confidence level, and SD_{blank} is the standard deviation of the blank measures; n = 5

Table S6. Levels (ng/g) of 4-OH-PCB52 and 4,4′-diOH-PCB52 in lung tissue for each exposure group[1]. Values are presented as the mean ± standard deviation.

**Analyte**	**Low 1%**	**Medium 5%**	**High 10%**
4-OH-PCB52	<LOD	17 ± 4	25 ± 7
4,4′-diOH-PCB52	<LOD	<LOD	<LOD

[1] Low 1%, *n* = 4; Medium 5%, *n* = 5; high 10%, *n* = 5

Table S7. Lung proteins significantly altered by medium 5% 4-OH-PCB52 exposure.

UniProt ID	Protein name	Protein ID	Medium vs. Sham	
			Log2(FC)[1]	FDR[2]
A0A8L2R3H7	C-type lectin domain family 2, member G	Clec2g	8.062	1.33E-12
Q6AYP5	Cell adhesion molecule 1	Cadm1	6.195	1.31E-11
A6J1U8	TP53 regulated inhibitor of apoptosis 1	Triap1	5.128	1.88E-10
A6H9R6	Serine and arginine rich splicing factor 7	Srsf7	5.098	1.31E-11
A0A8I6AXD4	USP6 N-terminal like	Usp6nl	4.534	1.31E-11
A0A140TAH1	Hepatocyte growth factor-regulated tyrosine kinase substrate	Hgs	4.435	1.13E-09
A6KKD7	Gene model 1960, isoform CRA_d	Gm1960	4.336	1.31E-11
F7FGN9	RSA-14-44	RSA-14-44	4.253	1.67E-08
A0A8I5Y7J0	Glycophorin A	Gypa	4.101	1.41E-06
E9PT44	Nuclear receptor subfamily 3 group C, member 1	Nr3c1	3.974	2.31E-09
O35370	Thrombomodulin	Thbd	3.693	1.60E-10
A0A8L2R247	Protein kinase 3	Pkn3	3.531	9.89E-08
Q6AXM6	Intercellular adhesion molecule 2	Icam2	3.467	1.42E-09
A0JPM9	Eukaryotic translation initiation factor 3, subunit J	Eif3j	3.339	9.71E-09
O88514	Degensin beta 4A	Defb4	3.301	6.54E-07
M0RDY6	Crystallin beta-gamma domain containing 3	Crybg3	3.196	8.97E-09
D3ZYS7	G3BP stress granule assembly factor 1	G3bp1	3.113	1.67E-08
A0A8I6A3F1	Cytochrome c oxidase assembly factor 5	Coa5	3.026	2.18E-09
A0A8I6AMP9	Carbonic anhydrase 2	Car2	3.009	1.03E-08
A0A8I5ZPF0	Haptoglobin	Hp	2.767	9.89E-08
A0A0G2K4W2	Transcription factor BTF3	Btf3l5	2.758	4.26E-08

UniProt ID	Protein name	Protein ID	Medium vs. Sham	
			Log2(FC)[1]	FDR[2]
A0A0G2K0Q8	Sushi domain containing 2	Susd2	2.757	8.97E-09
Q9JJ54	Heterogeneous nuclear ribonucleoprotein D	Hnrnpd	2.703	7.25E-09
B0K010	Thioredoxin domain-containing protein 17	Txndc17	2.671	2.54E-08
A0A0G2K0I6	Cadherin 5	Cdh5	2.659	4.36E-09
A0A0G2JTH4	CD47 molecule	Cd47	2.586	1.98E-07
A0A8I6AL15	Endothelin-converting enzyme 1	Ece1	2.382	2.25E-07
A0A8I6ANC4	Coronin 1C	Coro1c	2.376	1.27E-04
Q5PQU1	Kininogen 2	Kng2	2.271	1.26E-08
P31211	Serpin family A member 6	Serpina6	2.221	4.48E-07
A0A8I5ZYY0	H2A clustered histone 14	H2ac10	2.207	2.31E-04
A0A8I6A7X5	Cytochrome b5 type A	Cyb5a	2.166	3.81E-07
A0A8I6A5S9	Acidic leucine-rich nuclear phosphoprotein 32 family member A	Anp32a	2.141	1.41E-06
A0A8I6ADH4	Palladin-like 1	Palld	2.137	1.34E-07
A0A8I6AIG7	Transmembrane emp24 domain-containing protein 10	Tmed10	2.133	2.02E-05
A0A0G2K8C1	Nephronectin	Npnt	2.120	2.02E-05
A0A8L2RAD9	Tubulin alpha chain 1B	Tuba1b	2.120	6.51E-08
Q9ET61	CD93 molecule	Cd93	2.113	3.96E-06
Q9JM80	Phosphoprotein associated with glycosphingolipid-enriched microdomains 1	Pag1	2.111	4.53E-07
P25113	Phosphoglycerate mutase 1	Pgam1	2.083	1.97E-05
A0A8I6G5P0	Eukaryotic translation initiation factor 2 subunit 2	Eif2s2	2.009	3.19E-05
A0A8L2Q8D7	Podocalyxin	Podxl	1.960	1.63E-05
A6I9B9	Coronin 1A	Coro1a	1.945	5.12E-06

UniProt ID	Protein name	Protein ID	Medium vs. Sham	
			Log2(FC)[1]	FDR[2]
B0BNN3	Carbonic anhydrase 1	Ca1	1.940	1.61E-05
A0A8L2PZR6	Allograft inflammatory factor 1	Aif1	1.930	1.19E-05
A0A8I6ACN3	ADP-ribosylation factor (ARF) interacting protein 1	Arfip1	1.879	1.34E-07
A6HVR8	Ribosomal protein L34	Rpl34	1.860	1.79E-05
A0A0G2KB42	Clusterin	Clu	1.765	2.22E-06
A0A8I5ZKF7	Annexin A1	Anxa1	1.761	4.00E-06
A6IW22	Pleckstrin homology domain containing A2	Plekha2	1.720	2.12E-04
A0A0G2JUF5	Surfactant protein D	Sftpd	1.690	8.09E-07
A0A0G2K2B8	Tyrosine 3-monooxygenase/tryptophan 5-monooxygenase activation protein, eta	Ywhah	1.672	4.61E-03
A0A8I6ALE5	Translationally-controlled tumor protein 1 like 1	Tpt1l1	1.669	4.58E-07
Q63377	ATPase Na+/K+ transporting subunit beta-3	Atp1b3	1.645	8.63E-07
Q5GAM3	Eosinophi-associated ribonuclease A family member 1 like 1	Ear1l1	1.626	4.53E-04
Q5M7V8	Thyroid hormone receptor-associated protein 3	Thrap3	1.617	1.18E-06
A6JUY2	NADH dehydrogenase [ubiquinone] iron-sulfur protein 6, mitochondrial	Ndufs6	1.608	3.35E-07
A6HZL1	FKBP prolyl isomerase 2	Fkbp2	1.605	1.31E-05
F1M7Q7	OTU deubiquitinase 4	Otud4	1.573	3.96E-06
Q6P3E1	Ribosomal protein S16	Rps16	1.571	1.10E-06
A0A8I6A949	Beta-2-microglobulin	B2m	1.570	2.39E-06
A6J409	Coatomer subunit delta	Arcn1	1.547	3.81E-07
A0A8I5ZUT6	Ankyrin 3	Ank3	1.535	1.10E-06
A0A8I5ZR43	Interferon regulatory factor 2 binding protein 2	Irf2bp2	1.512	2.35E-05
A0A8L2R1R3	Parathymosin	Ptms	1.505	6.87E-04
A0A8I5YCL6	LIM domain containing 2	Limd2	1.406	2.97E-06

UniProt ID	Protein name	Protein ID	Medium vs. Sham	
			Log2(FC)[1]	FDR[2]
A0A8I6G659	H2A.Z variant histone 2	H2az2	1.404	2.75E-03
A6IGL8	Tetraspanin 8	Tspan8	1.387	4.96E-03
A0A8I5ZNH9	Septin 9	Septin9	1.373	1.18E-06
A0A8L2QZI9	Occludin	Ocln	1.334	2.12E-04
A0A8I6GKQ5	FKBP prolyl isomerase 3	Fkbp3	1.312	5.12E-06
G3V7Q4	Protein tyrosine phosphatase non-receptor type 12	Ptpn12	1.262	7.56E-06
Q5I034	Protein CUSTOS	Custos	1.231	9.28E-06
P19944	Ribosomal protein P1	Rplp1	1.228	3.76E-03
A0A8I6AHK0	Periostin	Postn	1.227	6.70E-05
A0A8I5ZZQ2	Prostaglandin E synthase 3	Ptges3	1.192	4.25E-03
B2RYX1	Ubiquinol-cytochrome c reductase, complex II subunit X	Uqcr10	1.172	2.79E-04
Q80YV8	C-X-C motif chemokine ligand 12	Cxcl12	1.171	2.00E-04
A0A8I6AKB2	Profilin 1	Pfn1	1.169	2.18E-03
A6IY70	Calreticulin	Calr	1.167	4.15E-03
M0R6L4	Ribosomal protein S3A	Rps3a	1.150	2.94E-04
P68255	Tyrosine 3-monooxygenase/tryptophan 5-monooxygenase activation protein, theta	Ywhaq	1.118	1.32E-04
A0A8I6AE98	Nucleoside diphosphate kinase 1	Nme1	1.117	1.77E-03
M0R9L0	Nascent polypeptide associated complex subunit alpha	Naca	1.100	4.57E-05
Q7TPJ0	Signal sequence receptor subunit 1	Ssr1	1.079	4.75E-04
A0A8I6G776	Ribosomal protein L19	Rpl19	1.077	5.82E-04
B1WC84	Canopy 4	Cnpy4	1.053	4.48E-03
A0A8I5ZZZ2	Serpin family A member 4	Serpina4	1.044	1.63E-03
A6HWS3	Zinc finger Ran-binding domain-containing protein 2	Zranb2	1.027	7.58E-05

UniProt ID	Protein name	Protein ID	Medium vs. Sham	
			Log2(FC)[1]	FDR[2]
A6IQS3	Activated leukocyte cell adhesion molecule	Alcam	1.019	6.37E-04
A0A0H2UHQ1	Ribosomal protein L17	Rpl17	1.019	6.64E-03
A6IEC5	Calumenin	Calu	1.016	2.67E-04
A0A8I5ZZF1	Ribosomal protein L22	Rpl22	1.010	1.70E-03
A6I6G9	Serpin family H member 1	Serpinh1	1.001	2.17E-04
Q6AY96	General transcription factor IIF subunit 1	Gtf2f1	-0.992	1.53E-04
Q9WVA1	Translocase of inner mitochondrial membrane 8A	Timm8a	-0.997	3.84E-04
A0A8I5ZS53	Host cell factor C1	Hcfc1	-1.010	3.76E-02
A0A8I6ALM9	Protein tyrosine kinase 7	Ptk7	-1.017	2.37E-05
A6J1E8	Endoplasmic reticulum resident protein 29	Erp29	-1.022	2.94E-04
A0A8L2QBW2	Cytochrome c oxidase subunit 5B, mitochondrial	Cox5b	-1.024	5.42E-05
A0A8L2QJB2	Ribosomal protein L11	Rpl11	-1.035	6.91E-04
P27321	Calpastatin	Cast	-1.038	2.79E-03
A0A8I6AUS6	Purkinje cell protein 4-like 1	Pcp4l1	-1.046	1.41E-05
B0BN81	Ribosomal protein S5	Rps5	-1.055	4.25E-03
A0A8I5Y758	CDC42 effector protein (Rho GTPase binding) 4, isoform CRA_a	Cdc42ep4	-1.055	4.17E-04
A0A8L2R0X2	Fibulin 5	Fbln5	-1.069	1.93E-03
Q5D059	Heterogeneous nuclear ribonucleoprotein K	Hnrnpk	-1.087	2.42E-04
Q6MG48	Proline rich coiled-coil 2A	Prrc2a	-1.093	5.21E-04
P52944	PDZ and LIM domain protein 1	Pdlim5	-1.104	2.34E-04
A0A8I6GF21	Coiled-coil domain containing 50	Ccdc50	-1.104	1.26E-05
B0BMS8	Myosin regulatory light polypeptide 9	Myl9	-1.112	4.14E-04
D4AB03	Family with sequence similarity 120, member A	Fam120a	-1.115	2.37E-05

UniProt ID	Protein name	Protein ID	Medium vs. Sham	
			Log2(FC)[1]	FDR[2]
F7FIS4	Tropomyosin 1	Tpi1	-1.139	4.46E-03
P62076	Translocase of inner mitochondrial membrane 13	Timm13	-1.174	1.12E-04
A0A8I6AFQ2	Tight junction protein 1	Tjp1	-1.175	8.71E-05
O88797	Disabled homolog 2	Dab2	-1.179	8.86E-05
A6J8U6	Basal cell adhesion molecule, isoform CRA_a	Bcam	-1.187	2.90E-05
A6HEB4	Ribosomal protein L30	Rpl30	-1.198	3.93E-06
F1LW91	Nuclear mitotic apparatus protein 1	Numa1	-1.210	7.52E-04
P02771	Albumin	Alb	-1.232	1.03E-02
Q923W4	Hepatoma-derived growth factor-related protein 3	Hdgfl3	-1.251	9.72E-05
A0A8L2QEH3	Ribosomal protein S19	Rps19	-1.256	1.88E-05
A0A0G2K4X8	S-phase kinase-associated protein 1	Skp1	-1.266	1.18E-05
A6IF31	Serine protease 1	Prss1	-1.272	2.92E-04
A0A8I6A790	Chloride intracellular channel protein 1	Clic1	-1.283	4.14E-04
P32038	Complement factor D	Cfd	-1.284	1.73E-05
A6KD00	Heterogeneous nuclear ribonucleoprotein A1	Hnrnpa1	-1.295	3.78E-04
A0A8I6GFT9	Formin binding protein 1	Fnbp1	-1.318	4.01E-05
A6I828	Lymphatic vessel endothelial hyaluronan receptor 1	Lyve1	-1.319	1.19E-05
A6KDU1	Thioredoxin 1	Txn1	-1.331	4.07E-05
A6KSD6	RCG42490, isoform CRA_b	RCG42490	-1.349	2.53E-04
A6JXV7	Dynein light intermediate chain	Dync1li2	-1.381	1.69E-04
A0A8I5ZMI8	Keratin, type II cytoskeletal 6A-like	LOC120093742	-1.387	1.37E-03
P18211	Rano class II histocompatibility antigen, D-1 beta chain	RT1-Db1	-1.423	2.34E-04
A0A8I5ZLU6	Afadin, adherens junction formation factor	Afdn	-1.434	1.02E-04

UniProt ID	Protein name	Protein ID	Medium vs. Sham	
			Log2(FC)[1]	FDR[2]
A0A8I6G597	Paralemmin 1	Palm	-1.463	1.20E-05
A6JQC5	Spectrin beta chain 1	Sptbn1	-1.464	1.39E-05
A0A8I5ZVN9	Ribosomal protein S11	Rps11	-1.478	7.56E-06
D4A2F2	Par-6 family cell polarity regulator beta	Pard6b	-1.489	2.52E-05
A6KAN9	MacroH2A.1 histone	Macroh2a1	-1.493	1.59E-06
A0A8I6ASE1	Antioxidant 1 cooper chaperone	Atox1	-1.507	9.77E-07
Q63575	Trans golgi network (TGN) specific integral membrane protein 41	Tgn41	-1.543	1.22E-05
P04166	Cytochrome b5 type B	Cyb5b	-1.546	4.98E-06
P62634	CCHC-type zinc finger nucleic acid binding protein	Cnbp	-1.546	5.55E-07
A6K8P9	Cold-inducible RNA-binding protein	Cirbp	-1.550	3.88E-05
A6KSI5	PYM homolog 1, exon junction complex associated factor	Pym1	-1.553	2.42E-06
A0A8I5Y0Q3	Family with sequence similarity 107, member B	Fam107b	-1.568	5.59E-05
A6JMG3	STING ER exit protein 1	Steep1	-1.590	4.38E-05
O35796	Complement component 1 Q subcomponent-binding protein, mitochondrial	C1qbp	-1.603	1.88E-04
A0A8I6G5X8	Heterogeneous nuclear ribonucleoprotein H1	Hnrnph1	-1.624	2.48E-05
A6K4W2	CGG triplet repeat binding protein 1	Cggbp1	-1.633	9.74E-07
A6HRN5	Alpha-1-B glycoprotein, isoform CRA_b	A1bg	-1.640	2.02E-05
A0A8I6AKF5	Synaptopodin	Synpo	-1.658	3.05E-06
F7F635	Alpha hemoglobin stabilizing protein	Ahsp	-1.663	6.26E-04
A6JMF3	Progesterone receptor membrane component 1	Pgrmc1	-1.681	1.14E-05

UniProt ID	Protein name	Protein ID	Medium vs. Sham	
			Log2(FC)[1]	FDR[2]
B0K013	Cytochrome c oxidase assembly factor 4	Coa4	-1.706	6.38E-06
A0A8L2QHS0	Ras interacting protein 1	Rasip1	-1.708	2.25E-07
Q5BKC3	Parkinsonism associated deglycase	Park7	-1.725	3.96E-06
Q9UFG5	Chromosome 19 open reading frame 25	C19orf25	-1.742	9.48E-06
G3V843	Coagulation factor II, thrombin	F2	-1.746	1.43E-06
A0A8L2UMW7	Parvalbumin	Pvalb	-1.759	1.33E-05
A0A8L2QE45	Craniofacial development protein 1	Cfdp1	-1.836	3.10E-07
A6JJQ5	Ribosomal protein L10A	Rpl10a	-1.845	2.06E-06
A0A8I6A5G9	Ganglioside GM2 activator	Gm2a	-1.895	1.98E-07
D3ZGK7	Carboxylic ester hydrolase 1C	Ces1c	-1.974	3.29E-04
A0A8I6AEU6	Ribosomal protein S8	Rps8	-2.012	2.60E-06
D4A2W1	Ectonucleotide pyrophosphate/phosphodiesterase 4	Enpp4	-2.016	3.81E-05
A6I5T3	Integrin alpha 1, isoform CRA_b	Itga1	-2.020	9.99E-07
Q63610	Tropomyosin 3	Tpm3	-2.052	3.53E-06
A0A0G2JZ52	Heterogeneous nuclear ribonucleoprotein U	Hnrnpu	-2.082	1.40E-08
A6JHQ8	STE20 like kinase	Slk	-2.148	1.05E-07
A0A8I5ZVI9	RAN binding protein 3	Ranbp3	-2.155	2.17E-08
A0A0G2K3Z9	Peroxiredoxin-1	Prdx1l1	-2.194	6.61E-08
A6JS43	Histidine-rich glycoprotein	Hrg	-2.272	8.77E-07
A0A8I6AHB3	Lysosomal-associated membrane protein 2	Lamp2	-2.281	3.56E-07
A0A0G2K6Y9	Syntaxin 7	Stx7	-2.373	5.14E-08
Q02356	AMP deaminase 2	Ampd2	-2.422	2.17E-08

UniProt ID	Protein name	Protein ID	Medium vs. Sham	
			Log2(FC)[1]	FDR[2]
A6HZZ1	Secretoglobin family 1A member 1	Scgb1a1	-2.548	2.42E-06
A0A8I6AMC9	C3 and PZP-like, alpha-2-macroglobulin domain containing 8	Cpamd8	-2.569	5.36E-06
P05942	S100 calcium binding protein A4	S100a4	-2.575	1.60E-06
D3ZV50	Ribosomal protein S91 like 1	Rps9l1	-2.640	5.41E-08
A0A8L2PYP2	C-reactive protein	Crp	-2.802	1.05E-07
A0A0G2K151	Apolipoprotein E	Apoe	-2.840	6.33E-08
A0A8I6GID8	Integrin subunit alpha 8	Itga8	-2.944	1.02E-09
F7FL28	Napsin A aspartic peptidase	Napsa	-2.966	2.07E-08
A6I4B3	C-type lectin domain family 3, member G	Clec3b	-3.090	1.34E-07
D4A636	IKAROS family zinc finger 4	Ikzf4	-3.285	3.45E-08
A0A8I6A7A5	ribosomal protein S24	Rps24	-3.436	4.36E-09
Q6AYD4	Endothelial cell-selective adhesion molecule	Esam	-3.546	1.59E-10
A0A8L2QDS2	Glutathione S-transferase mu 2	Gstm2	-4.052	4.68E-11
Q6P790	Ribosomal protein L6	Rpl6	-4.599	1.88E-10
A6J2G7	Ribosomal protein S26	Rps26	-4.631	3.67E-11
A6JS59	Transformer 2 beta homolog	Tra2b	-4.653	3.04E-10
F1M9N4	Muscleblind-like splicing regulator 1	Mbnl1	-6.625	1.51E-12

[1]Log2 of fold-change of ±1.0

[2]False discovery rate represents the adjusted *P*-value of 0.05

Table S8. KEGG enrichment pathways associated with medium 5% 4-OH-PCB52 exposure.

**KEGG pathway**	**Count in network**	**Strength**	**FDR[1]**
Ribosome	16/158	1.09	3.30E-10
Phagosome	8/149	0.82	0.007
Cell adhesion molecules	8/147	0.82	0.007
Leukocyte transendothelial migration	6/109	0.83	0.022
Tight junction	7/146	0.77	0.022
Regulation of actin cytoskeleton	8/199	0.69	0.022
Complement and coagulation cascades	5/78	0.89	0.022

[1]False discovery rate represents the adjusted *P*-value of 0.05

Table S9. Lung proteins significantly altered by high 10% 4-OH-PCB52 exposure.

UniProt ID	Protein name	Protein ID	High vs. Sham	
			Log2(FC)[1]	FDR[2]
A0A8L2R3H7	C-type lectin domain family 2, member G	Clec2g	7.976	1.19E-11
Q6AYP5	Cell adhesion molecule 1	Cadm1	6.046	8.85E-10
A6J1U8	TP53 regulated inhibitor of apoptosis 1	Triap1	5.346	3.93E-09
A6H9R6	Serine and arginine rich splicing factor 7	Srsf7	4.913	1.13E-08
A0A8I6AXD4	USP6 N-terminal like	Usp6nl	4.361	9.34E-09
E9PT44	Nuclear receptor subfamily 3 group C member 1	Nr3c1	4.244	1.47E-08
A6KKD7	Gene model 1960, isoform CRA_d	Gm1960	4.121	1.47E-08
A0A140TAH1	Hepatocyte growth factor-regulated tyrosine kinase substrate	Hgs	4.101	3.57E-07
A0A8I5Y7J0	Glycophorin A	Gypa	3.869	1.93E-05
F7FGN9	RSA-14-44	RSA-14-44	3.834	3.52E-08
O35370	Thrombomodulin	Thbd	3.558	5.63E-08
M0RDY6	Crystallin beta-gamma domain containing 3	Crybg3	3.506	1.48E-05
A0A8L2R247	Protein kinase 3	Pkn3	3.478	4.91E-07
O88514	Degensin beta 4A	Defb4	3.460	4.44E-05
Q6AXM6	Intercellular adhesion molecule 2	Icam2	3.370	2.24E-07
A0A8I6A3F1	Cytochrome c oxidase assembly factor 5	Coa5	3.078	3.74E-07
A0JPM9	Eukaryotic translation initiation factor 3 subunit J	Eif3j	3.076	7.82E-07
D3ZYS7	G3BP stress granule assembly factor 1	G3bp1	2.828	1.96E-06
A0A8I6AMP9	Carbonic anhydrase 2	Car2	2.634	1.89E-06
A0A8I5ZPF0	Haptoglobin	Hp	2.558	3.61E-06
A0A0G2JTH4	CD47 molecule	Cd47	2.550	1.93E-05
A0A0G2K4W2	Transcription factor BTF3	Btf3l5	2.514	1.74E-06

UniProt ID	Protein name	Protein ID	High vs. Sham	
			Log2(FC)[1]	FDR[2]
A0A0G2K0Q8	Sushi domain containing 2	Susd2	2.483	3.74E-07
B0K010	Thioredoxin domain-containing protein 17	Txndc17	2.469	1.28E-07
A0A0G2K0I6	Cadherin 5	Cdh5	2.464	1.42E-06
Q9JJ54	Heterogeneous nuclear ribonucleoprotein D	Hnrnpd	2.262	4.49E-08
A0A8I5ZYY0	H2A clustered histone 14	H2ac10	2.256	2.82E-04
A0A8I6AL15	Endothelin-converting enzyme 1	Ece1	2.201	1.03E-05
A0A8I6ADH4	Palladin-like 1	Palld	2.197	3.73E-05
Q5PQU1	Kininogen 2	Kng2	2.167	7.55E-06
A0A0G2K8C1	Nephronectin	Npnt	2.129	4.59E-05
Q9JM80	Phosphoprotein associated with glycosphingolipid-enriched microdomains 1	Pag1	2.115	1.29E-05
A0A8I6A5S9	Acidic leucine-rich nuclear phosphoprotein 32 family member A	Anp32a	2.048	4.71E-06
A0A8I6A7X5	Cytochrome b5 type A	Cyb5a	2.002	7.72E-06
P31211	Serpin family A member 6	Serpina6	1.915	1.56E-05
A0A8L2RAD	Tubulin alpha chain 1B	Tuba1b	1.886	9.47E-06
A0A8L2Q8D7	Podocalyxin	Podxl	1.883	7.43E-05
Q9ET61	CD93 molecule	Cd93	1.877	1.56E-04
A0A8I6G5P0	Eukaryotic translation initiation factor 2 subunit 2	Eif2s2	1.871	1.15E-04
A6HVR8	Ribosomal protein L34	Rpl34	1.869	1.11E-04
P25113	Phosphoglycerate mutase 1	Pgam1	1.840	3.43E-04
A0A8I6ANC4	Coronin 1C	Coro1c	1.829	4.01E-04
A0A8I6AIG7	Transmembrane emp24 domain-containing protein 10	Tmed10	1.807	1.40E-04
A0A8I6ACN3	ADP-ribosylation factor (ARF) interacting protein 1	Arfip1	1.791	7.72E-06
Q5GAM3	Eosinophi-associated ribonuclease A family member 1 like 1	Ear1l1	1.725	3.57E-05
A0A0G2KB42	Clusterin	Clu	1.694	2.71E-05

UniProt ID	Protein name	Protein ID	High vs. Sham	
			Log2(FC)[1]	FDR[2]
A0A0G2K2B8	Tyrosine 3-monooxygenase/tryptophan 5-monooxygenase activation protein, eta	Ywhah	1.685	8.48E-03
A6IW22	Pleckstrin homology domain containing A2	Plekha2	1.685	8.32E-04
A0A8L2PZR6	Allograft inflammatory factor 1	Aif1	1.644	1.42E-04
A0A8I5ZKF7	Annexin A1	Anxa1	1.642	9.50E-05
Q63377	ATPase Na+/K+ transporting subunit beta-3	Atp1b3	1.620	7.72E-06
A6JUY2	NADH dehydrogenase [ubiquinone] iron-sulfur protein 6, mitochondrial	Ndufs6	1.617	1.68E-05
A6I9B9	Coronin 1A	Coro1a	1.608	1.11E-04
Q5M7V8	Thyroid hormone receptor-associated protein 3	Thrap3	1.579	1.83E-05
A6HZL1	FKBP prolyl isomerase 2	Fkbp2	1.539	1.43E-05
A0A8I6ALE5	Translationally-controlled tumor protein 1 like 1	Tpt1l1	1.527	1.35E-06
A0A8I5ZUT6	Ankyrin 3	Ank3	1.515	1.93E-05
F1M7Q7	OTU deubiquitinase 4	Otud4	1.466	3.52E-05
P19944	Ribosomal protein P1	Rplp1	1.463	5.84E-03
B0BNN3	Carbonic anhydrase 1	Ca1	1.438	3.10E-04
Q6P3E1	Ribosomal protein S16	Rps16	1.408	1.46E-05
A0A8I5ZR43	Interferon regulatory factor 2 binding protein 2	Irf2bp2	1.400	2.70E-04
A0A8I6G659	H2A.Z variant histone 2	H2az2	1.366	1.58E-03
A6J409	Coatomer subunit delta	Arcn1	1.353	1.59E-04
A0A0G2JUF5	Surfactant protein D	Sftpd	1.348	2.78E-05
A0A8I5YCL6	LIM domain containing 2	Limd2	1.289	1.11E-04
A0A8L2QZI9	Occludin	Ocln	1.270	8.05E-04
A0A8L2R1R3	Parathymosin	Ptms	1.247	4.19E-04
A0A8I5ZNH9	Septin 9	Septin9	1.245	7.10E-05
Q5I034	Protein CUSTOS	Custos	1.220	1.37E-04
A0A8I6GKQ5	FKBP prolyl isomerase 3	Fkbp3	1.211	1.40E-04
A6IGL8	Tetraspanin 8	Tspan8	1.192	5.79E-03

UniProt ID	Protein name	Protein ID	High vs. Sham	
			Log2(FC)[1]	FDR[2]
A0A8I6A949	Beta-2-microglobulin	B2m	1.137	5.27E-04
G3V7Q4	Protein tyrosine phosphatase non-receptor type 12	Ptpn12	1.135	1.01E-04
A0A8I6AHK0	Periostin	Postn	1.123	3.22E-04
A0A8I5ZZZ2	Serpin family A member 4	Serpina4	1.112	4.98E-04
Q7TPJ0	Signal sequence receptor subunit 1	Ssr1	1.098	8.20E-04
A0A8I6G776	Ribosomal protein L19	Rpl19	1.061	4.61E-04
A6IQS3	Activated leukocyte cell adhesion molecule	Alcam	1.049	1.95E-03
B2RYX1	Ubiquinol-cytochrome c reductase, complex II subunit X	Uqcr10	1.048	2.43E-03
A0A8I5ZZQ2	Prostaglandin E synthase 3	Ptges3	1.043	9.60E-04
A6HWS3	Zinc finger Ran-binding domain-containing protein 2	Zranb2	1.025	5.04E-04
M0R9L0	Nascent polypeptide associated complex subunit alpha	Naca	1.016	2.58E-04
A0A8I6AE98	Nucleoside diphosphate kinase 1	Nme1	0.998	2.81E-04
B0BMS8	Myosin regulatory light polypeptide 9	Myl9	-0.993	3.51E-03
Q5D059	Heterogeneous nuclear ribonucleoprotein K	Hnrnpk	-0.994	7.74E-04
A6I828	Lymphatic vessel endothelial hyaluronan receptor 1	Lyve1	-0.999	9.47E-03
A6IF31	Serine protease 1	Prss1	-1.010	4.48E-03
A0A8I5ZS53	Host cell factor C1	Hcfc1	-1.026	1.93E-02
A0A8I6GFT9	Formin binding protein 1	Fnbp1	-1.036	5.27E-04
A0A8L2Q474	Small muscular protein	Smpx	-1.040	9.91E-03
A0A8I6GF21	Coiled-coil domain containing 50	Ccdc50	-1.047	1.50E-04
Q6MG48	Proline rich coiled-coil 2A	Prrc2a	-1.055	5.53E-04
Q5XI07	Lipoma-preferred partner homolog	Lpp	-1.055	8.98E-04

UniProt ID	Protein name	Protein ID	High vs. Sham	
			Log2(FC)[1]	FDR[2]
A6J1E8	Endoplasmic reticulum resident protein 29	Erp29	-1.058	1.01E-04
P32038	Complement factor D	Cfd	-1.073	8.16E-04
A0A8I6ALM9	Protein tyrosine kinase 7	Ptk7	-1.077	2.31E-04
P62076	Translocase of inner mitochondrial membrane 13	Timm13	-1.088	1.39E-04
D4A2F2	Par-6 family cell polarity regulator beta	Pard6b	-1.089	1.05E-02
A0A8L2QEH3	Ribosomal protein S19	Rps19	-1.101	5.34E-04
F1LW91	Nuclear mitotic apparatus protein 1	Numa1	-1.102	2.61E-03
A6J8U6	Basal cell adhesion molecule, isoform CRA_a	Bcam	-1.135	2.17E-04
O88797	Disabled homolog 2	Dab2	-1.138	3.10E-04
A0A8I5ZJ94	Ankyrin 1	Ank1	-1.139	2.84E-04
P14046	Alpha-1-inhibitor 3	A1i3	-1.141	1.38E-03
A6JMG3	STING ER exit protein 1	Steep1	-1.160	2.43E-03
A0A8I6AFQ2	Tight junction protein 1	Tjp1	-1.180	1.87E-04
A0A0G2K4X8	S-phase kinase-associated protein 1	Skp1	-1.188	8.21E-04
A0A8I6A790	Chloride intracellular channel protein 1	Clic1	-1.206	2.69E-03
A6KSD6	RCG42490, isoform CRA_b	RCG42490	-1.228	4.59E-04
A0A8I6ASE1	Antioxidant 1 cooper chaperone	Atox1	-1.242	1.16E-03
O35796	Complement component 1 Q subcomponent-binding protein, mitochondrial	C1qbp	-1.242	1.83E-02
A0A8L2UMW7	Parvalbumin	Pvalb	-1.264	1.12E-02
A0A8I5ZLU6	Afadin, adherens junction formation factor	Afdn	-1.265	7.30E-04
A6K8P9	Cold-inducible RNA-binding protein	Cirbp	-1.267	3.94E-03
A6KDU1	Thioredoxin 1	Txn1	-1.268	4.77E-04
A0A8I6G5X8	Heterogeneous nuclear ribonucleoprotein H1	Hnrnph1	-1.274	3.51E-03

UniProt ID	Protein name	Protein ID	High vs. Sham	
			Log2(FC)[1]	FDR[2]
P18211	Rano class II histocompatibility antigen, D-1 beta chain	RT1-Db1	-1.274	4.73E-04
A6JXV7	Dynein light intermediate chain	Dync1li2	-1.279	4.84E-04
A6JQC5	Spectrin beta chain 1	Sptbn1	-1.310	6.46E-05
A6KAN9	MacroH2A.1 histone	Macroh2a1	-1.344	2.59E-05
Q63575	Trans golgi network specific integral membrane protein 41	Tgn41	-1.345	2.14E-03
F7FIS4	Tropomyosin 1	Tpi1	-1.354	9.28E-04
A6KD00	Heterogeneous nuclear ribonucleoprotein A1	Hnrnpa1	-1.365	4.27E-04
A0A8I5ZVN9	Ribosomal protein S11	Rps11	-1.371	6.52E-05
B0K013	Cytochrome c oxidase assembly factor 4	Coa4	-1.373	3.79E-04
A0A8I5ZMI8	Keratin, type II cytoskeletal 6A-like	LOC120093742	-1.376	1.86E-03
P62634	CCHC-type zinc finger nucleic acid binding protein	Cnbp	-1.401	2.25E-04
P04166	Cytochrome b5 type B	Cyb5b	-1.408	6.11E-05
A0A8I5Y0Q3	Family with sequence similarity 107, member B	Fam107b	-1.415	2.34E-04
A6KSI5	PYM homolog 1, exon junction complex associated factor	Pym1	-1.430	3.52E-05
A0A8I6G597	Paralemmin 1	Palm	-1.442	1.01E-04
A0A8I6AKF5	Synaptopodin	Synpo	-1.506	1.82E-05
A0A8L2QHS0	Ras interacting protein 1	Rasip1	-1.548	1.95E-05
A0A8L2QE45	Craniofacial development protein 1	Cfdp1	-1.550	6.34E-05
Q5BKC3	Parkinsonism associated deglycase	Park7	-1.552	5.88E-05
A6K4W2	CGG triplet repeat binding protein 1	Cggbp1	-1.565	1.38E-05
Q9UFG5	Chromosome 19 open reading frame 25	C19orf25	-1.568	2.67E-04

UniProt ID	Protein name	Protein ID	High vs. Sham	
			Log2(FC)[1]	FDR[2]
A6JMF3	Progesterone receptor membrane component 1	Pgrmc1	-1.596	5.83E-05
G3V843	Coagulation factor II, thrombin	F2	-1.633	2.33E-05
A6HRN5	Alpha-1-B glycoprotein, isoform CRA_b	A1bg	-1.645	2.34E-04
D4A2W1	Ectonucleotide pyrophosphate/phosphodiesterase 4	Enpp4	-1.692	3.16E-03
A6JJQ5	Ribosomal protein L10A	Rpl10a	-1.695	2.58E-04
A0A8I6A5G9	Ganglioside GM2 activator	Gm2a	-1.704	3.38E-05
A0A8I6AEU6	Ribosomal protein S8	Rps8	-1.749	2.56E-04
F7F635	Alpha hemoglobin stabilizing protein	Ahsp	-1.754	4.45E-04
A0A8I6GID8	Integrin subunit alpha 8	Itga1	-1.772	6.34E-05
A0A8I6AHB3	Lysosomal-associated membrane protein 2	Lamp2	-1.781	1.71E-04
A0A8I5ZVI9	RAN binding protein 3	Ranbp3	-1.842	1.68E-05
A0A0G2JZ52	Heterogeneous nuclear ribonucleoprotein U	Hnrnpu	-1.856	2.45E-05
D3ZGK7	Carboxylic ester hydrolase 1C	Ces1c	-1.858	1.90E-03
A6JHQ8	STE20 like kinase	Slk	-1.945	2.59E-05
A6JS43	Histidine-rich glycoprotein	Hrg	-1.988	1.05E-04
A0A0G2K6Y9	Syntaxin 7	Stx7	-2.067	3.84E-05
Q02356	AMP deaminase 2	Ampd2	-2.090	1.10E-04
Q63610	Tropomyosin 3	Tpm3	-2.178	4.30E-05
A6HZZ1	Secretoglobin family 1A member 1	Scgb1a1	-2.215	6.34E-05
A0A0G2K3Z9	Peroxiredoxin-1	Prdx1l1	-2.221	2.16E-04
P05942	S100 calcium binding protein A4	S100a4	-2.303	1.87E-04
F7FL28	Napsin A aspartic peptidase	Napsa	-2.423	8.79E-04
D3ZV50	Ribosomal protein S91 like 1	Rps9l1	-2.429	3.15E-05
A6I4B3	C-type lectin domain family 3, member B	Clec3b	-2.526	7.91E-04
A0A8L2PYP2	C-reactive protein	Crp	-2.566	3.15E-05
A0A8I6GID8	Integrin subunit alpha 8	Itga8	-2.616	6.34E-05

UniProt ID	Protein name	Protein ID	High vs. Sham	
			Log2(FC)[1]	FDR[2]
A0A8I6AMC9	C3 and PZP-like, alpha-2-macroglobulin domain containing 8	Cpamd8	-2.645	3.34E-04
D4A636	IKAROS family zinc finger 4	Ikzf4	-2.725	5.06E-04
A0A8I6A7A5	Ribosomal protein S24	Rps24	-2.773	7.12E-04
A0A0G2K151	Apolipoprotein E	Apoe	-2.799	1.20E-05
Q6AYD4	Endothelial cell-selective adhesion molecule	Esam	-3.081	7.67E-05
A0A8L2QDS2	Glutathione S-transferase mu 2	Gstm2	-3.397	4.91E-04
A6J2G7	Ribosomal protein S26	Rps26	-3.607	1.51E-03
A6JS59	Transformer 2 beta homolog	Tra2b	-3.813	2.74E-04
Q6P790	Ribosomal protein L6	Rpl6	-3.994	8.88E-05
F1M9N4	Muscleblind-like splicing regulator 1	Mbnl1	-5.447	3.22E-04

[1]Log2 of fold-change of ± 1.0

[2]False discovery rate represents the adjusted *P*-value of 0.05

Table S10. KEGG enrichment pathways associated with high 10% 4-OH-PCB52 exposure.

**KEGG pathway**	**Count in network**	**Strength**	**FDR[1]**
Ribosome	11/158	0.98	1.35E-05
Complement and coagulation cascades	5/78	0.95	0.022
Cell adhesion molecules	7/146	0.82	0.020
Tight junction	7/147	0.82	0.020
Phagosome	7/149	0.81	0.020

[1]False discovery rate represents the adjusted *P*-value of 0.05

Table S11. Common lung proteins altered between medium 5% and high 10% exposure of 4-OH-PCB52.

UniProt ID	Protein name	Protein ID	Medium vs. Sham		High vs. Sham	
			Log2(FC)[1]	FDR[2]	Log2(FC)	FDR
A0A8L2R3H7	C-type lectin domain family 2, member G	Clec2g	8.063	1.33E-12	7.976	1.19E-11
Q6AYP5	Cell adhesion molecule 1	Cadm1	6.195	1.31E-11	6.046	8.85E-10
A6J1U8	TP53 regulated inhibitor of apoptosis 1	Triap1	5.128	1.88E-10	5.346	3.93E-09
A6H9R6	Serine and arginine rich splicing factor 7	Srsf7	5.098	1.31E-11	4.913	1.13E-08
A0A8I6AXD4	USP6 N-terminal like	Usp6nl	4.534	1.31E-11	4.361	9.34E-09
A0A140TAH1	Hepatocyte growth factor-regulated tyrosine kinase substrate	Hgs	4.435	1.13E-09	4.101	3.57E-07
A6KKD7	Gene model 1960, isoform CRA_d	Gm1960	4.336	1.31E-11	4.121	1.47E-08
F7FGN9	RSA-14-44	RSA-14-44	4.253	1.67E-08	3.834	3.52E-08
A0A8I5Y7J0	Glycophorin A	Gypa	4.101	1.41E-06	3.869	1.93E-05
E9PT44	Nuclear receptor subfamily 3 group C member 1	Nr3c1	3.974	2.31E-09	4.244	1.47E-08
O35370	Thrombomodulin	Thbd	3.693	1.59E-10	3.558	5.63E-08
A0A8L2R247	Protein kinase 3	Pkn3	3.531	9.89E-08	3.478	4.91E-07
Q6AXM6	Intercellular adhesion molecule 2	Icam2	3.467	1.42E-09	3.370	2.24E-07
A0JPM9	Eukaryotic translation	Eif3j	3.339	9.71E-09	3.076	7.82E-07

UniProt ID	Protein name	Protein ID	Medium vs. Sham		High vs. Sham	
			Log2(FC)[1]	FDR[2]	Log2(FC)	FDR
	initiation factor 3 subunit J					
O88514	Degensin beta 4A	Defb4	3.302	6.54E-07	3.460	4.44E-05
M0RDY6	Crystallin beta-gamma domain containing 3	Crybg3	3.196	8.97E-09	3.506	1.48E-05
D3ZYS7	G3BP stress granule assembly factor 1	G3bp1	3.113	1.67E-08	2.828	1.96E-06
A0A8I6A3F1	Cytochrome c oxidase assembly factor 5	Coa5	3.026	2.18E-09	3.078	3.74E-07
A0A8I6AMP9	Carbonic anhydrase 2	Car2	3.009	1.03E-08	2.634	1.89E-06
A0A8I5ZPF0	Haptoglobin	Hp	2.767	9.89E-08	2.558	3.61E-06
A0A0G2K4W2	Transcription factor BTF3	Btf3l5	2.758	4.26E-08	2.514	1.74E-06
A0A0G2K0Q8	Sushi domain containing 2	Susd2	2.757	8.97E-09	2.483	3.74E-07
Q9JJ54	Heterogeneous nuclear ribonucleoprotein D	Hnrnpd	2.704	7.25E-09	2.262	4.49E-08
B0K010	Thioredoxin domain-containing protein 17	Txndc17	2.671	2.54E-08	2.469	1.28E-07
A0A0G2K0I6	Cadherin 5	Cdh5	2.659	4.36E-09	2.464	1.42E-06
A0A0G2JTH	CD47 molecule	Cd47	2.586	1.98E-07	2.550	1.93E-05
A0A8I6AL15	Endothelin-converting enzyme 1	Ece1	2.382	2.25E-07	2.201	1.03E-05
A0A8I6ANC4	Coronin 1C	Coro1c	2.376	1.27E-04	1.829	4.01E-04

UniProt ID	Protein name	Protein ID	Medium vs. Sham		High vs. Sham	
			Log2(FC)[1]	FDR[2]	Log2(FC)	FDR
Q5PQU1	Kininogen 2	Kng2	2.271	1.26E-08	2.167	7.55E-06
P31211	Serpin family A member 6	Serpina6	2.221	4.48E-07	1.915	1.56E-05
A0A8I5ZYY0	H2A clustered histone 14	H2ac10	2.207	2.31E-04	2.256	2.82E-04
A0A8I6A7X5	Cytochrome b5 type A	Cyb5a	2.166	3.81E-07	2.002	7.72E-06
A0A8I6A5S9	Acidic leucine-rich nuclear phosphoprotein 32 family member A	Anp32a	2.141	1.41E-06	2.048	4.71E-06
A0A8I6ADH4	Palladin-like 1	Palld	2.137	1.34E-07	2.197	3.73E-05
A0A8I6AIG7	Transmembrane emp24 domain-containing protein 10	Tmed10	2.133	2.02E-05	1.807	1.40E-04
A0A0G2K8C1	Nephronectin	Npnt	2.120	2.02E-05	2.129	4.59E-05
A0A8L2RAD9	Tubulin alpha chain 1B	Tuba1b	2.120	6.51E-08	1.886	9.47E-06
Q9ET61	CD93 molecule	Cd93	2.113	3.96E-06	1.877	1.56E-04
Q9JM80	Phosphoprotein associated with glycosphingolipid-enriched microdomains 1	Pag1	2.111	4.53E-07	2.115	1.29E-05
P25113	Phosphoglycerate mutase 1	Pgam1	2.083	1.97E-05	1.840	3.43E-04
A0A8I6G5P0	Eukaryotic translation initiation factor 2 subunit 2	Eif2s2	2.009	3.19E-05	1.871	1.15E-04
A0A8L2Q8D7	Podocalyxin	Podxl	1.960	1.63E-05	1.883	7.43E-05

UniProt ID	Protein name	Protein ID	Medium vs. Sham		High vs. Sham	
			Log2(FC)[1]	FDR[2]	Log2(FC)	FDR
A6I9B9	Coronin 1A	Coro1a	1.945	5.12E-06	1.608	1.11E-04
B0BNN3	Carbonic anhydrase 1	Ca1	1.940	1.61E-05	1.438	3.10E-04
A0A8L2PZR6	Allograft inflammatory factor 1	Aif1	1.930	1.19E-05	1.644	1.42E-04
A0A8I6ACN3	ADP-ribosylation factor (ARF) interacting protein 1	Arfip1	1.879	1.34E-07	1.791	7.72E-06
A6HVR8	Ribosomal protein L34	Rpl34	1.860	1.79E-05	1.869	1.11E-04
A0A0G2KB42	Clusterin	Clu	1.765	2.22E-06	1.694	2.71E-05
A0A8I5ZKF7	Annexin A1	Anxa1	1.761	4.00E-06	1.642	9.50E-05
A6IW22	Pleckstrin homology domain containing A2	Plekha2	1.720	2.12E-04	1.685	8.32E-04
A0A0G2JUF5	Surfactant protein D	Sftpd	1.6898	8.09E-07	1.348	2.78E-05
A0A0G2K2B8	Tyrosine 3-monooxygenase/tryptophan 5-monooxygenase activation protein, eta	Ywhah	1.672	4.61E-03	1.685	8.48E-03
A0A8I6ALE5	Translationally-controlled tumor protein 1 like 1	Tpt1l1	1.669	4.58E-07	1.527	1.35E-06
Q63377	ATPase Na+/K+ transporting subunit beta-3	Atp1b3	1.645	8.63E-07	1.620	7.72E-06
Q5GAM3	Eosinophi-associated ribonuclease A	Ear1l1	1.626	4.53E-04	1.725	3.57E-05

UniProt ID	Protein name	Protein ID	Medium vs. Sham		High vs. Sham	
			Log2(FC)[1]	FDR[2]	Log2(FC)	FDR
	family member 1 like 1					
Q5M7V8	Thyroid hormone receptor-associated protein 3	Thrap3	1.617	1.18E-06	1.579	1.83E-05
A6JUY2	NADH dehydrogenase [ubiquinone] iron-sulfur protein 6, mitochondrial	Ndufs6	1.608	3.35E-07	1.617	1.68E-05
A6HZL1	FKBP prolyl isomerase 2	Fkbp2	1.605	1.31E-05	1.539	1.43E-05
F1M7Q7	OTU deubiquitinase 4	Otud4	1.573	3.96E-06	1.466	3.52E-05
Q6P3E1	Ribosomal protein S16	Rps16	1.571	1.10E-06	1.408	1.46E-05
A0A8I6A949	Beta-2-microglobulin	B2m	1.570	2.39E-06	1.137	5.27E-04
A6J409	Coatomer subunit delta	Arcn1	1.547	3.81E-07	1.353	1.59E-04
A0A8I5ZUT6	Ankyrin 3	Ank3	1.535	1.10E-06	1.515	1.93E-05
A0A8I5ZR43	Interferon regulatory factor 2 binding protein 2	Irf2bp2	1.512	2.35E-05	1.400	2.70E-04
A0A8L2R1R3	Parathymosin	Ptms	1.505	6.87E-04	1.247	4.19E-04
A0A8I5YCL6	LIM domain containing 2	Limd2	1.406	2.97E-06	1.289	1.11E-04
A0A8I6G659	H2A.Z variant histone 2	H2az2	1.404	2.75E-03	1.366	1.58E-03
A6IGL8	Tetraspanin 8	Tspan8	1.387	4.96E-03	1.192	5.79E-03
A0A8I5ZNH9	Septin 9	Septin9	1.373	1.18E-06	1.245	7.10E-05

UniProt ID	Protein name	Protein ID	Medium vs. Sham		High vs. Sham	
			Log2(FC)[1]	FDR[2]	Log2(FC)	FDR
A0A8L2QZI9	Occludin	Ocln	1.334	2.12E-04	1.270	8.05E-04
A0A8I6GKQ5	FKBP prolyl isomerase 3	Fkbp3	1.312	5.12E-06	1.211	1.40E-04
G3V7Q4	Protein tyrosine phosphatase non-receptor type 12	Ptpn12	1.262	7.56E-06	1.135	1.01E-04
Q5I034	Protein CUSTOS	Custos	1.231	9.28E-06	1.220	1.37E-04
P19944	Ribosomal protein P1	Rplp1	1.228	3.76E-03	1.463	5.84E-03
A0A8I6AHK0	Periostin	Postn	1.227	6.70E-05	1.123	3.22E-04
A0A8I5ZZQ2	Prostaglandin E synthase 3	Ptges3	1.1917	4.25E-03	1.043	9.60E-04
B2RYX1	Ubiquinol-cytochrome c reductase, complex II subunit X	Uqcr10	1.172	2.79E-04	1.048	2.43E-03
Q80YV8	C-X-C motif chemokine ligand 12	Cxcl12	1.171	2.00E-04	0.982	2.75E-03
A0A8I6AKB2	Profilin 1	Pfn1	1.169	2.18E-03	0.881	1.20E-02
A6IY70	Calreticulin	Calr	1.167	4.15E-03	0.730	1.54E-02
M0R6L4	Ribosomal protein S3A	RPS3A	1.150	2.94E-04	0.873	1.21E-03
P68255	Tyrosine 3-monooxygenase/tryptophan 5-monooxygenase activation protein, theta	Ywhaq	1.118	1.32E-04	0.936	8.42E-04

UniProt ID	Protein name	Protein ID	Medium vs. Sham		High vs. Sham	
			Log2(FC)[1]	FDR[2]	Log2(FC)	FDR
A0A8I6AE98	Nucleoside diphosphate kinase 1	Nme1	1.117	1.77E-03	0.998	2.81E-04
M0R9L0	Nascent polypeptide associated complex subunit alpha	Naca	1.100	4.57E-05	1.016	2.58E-04
Q7TPJ0	Signal sequence receptor subunit 1	Ssr1	1.079	4.75E-04	1.098	8.20E-04
A0A8I6G776	Ribosomal protein L19	Rpl19	1.077	5.82E-04	1.061	4.61E-04
B1WC84	Canopy 4	Cnpy4	1.053	4.48E-03	0.892	3.99E-03
A0A8I5ZZZ2	Serpin family A member 4	Serpina4	1.044	1.63E-03	1.112	4.98E-04
A6HWS3	Zinc finger Ran-binding domain-containing protein 2	Zranb2	1.027	7.58E-05	1.025	5.04E-04
A6IQS3	Activated leukocyte cell adhesion molecule	Alcam	1.019	6.37E-04	1.049	1.95E-03
A0A0H2UHQ1	Ribosomal protein L17	Rpl17	1.020	6.64E-03	0.946	5.60E-03
A6IEC5	Calumenin	Calu	1.016	2.67E-04	0.776	1.24E-03
A0A8I5ZZF1	Ribosomal protein L22	Rpl22	1.010	1.70E-03	0.912	3.13E-03
A6I6G9	Serpin family H member 1	Serpinh1	1.001	2.17E-04	0.713	3.18E-03
A0A8I5ZS53	Host cell factor C1	Hcfc1	-1.010	3.76E-02	-1.026	1.93E-02
A0A8I6ALM9	Protein tyrosine kinase 7	Ptk7	-1.017	2.37E-05	-1.077	2.31E-04

UniProt ID	Protein name	Protein ID	Medium vs. Sham		High vs. Sham	
			Log2(FC)[1]	FDR[2]	Log2(FC)	FDR
A6J1E8	Endoplasmic reticulum resident protein 29	Erp29	-1.022	2.94E-04	-1.058	1.01E-04
A0A8L2QBW2	Cytochrome c oxidase subunit 5B, mitochondrial	Cox5b	-1.024	5.42E-05	-0.940	4.84E-04
A0A8L2QJB2	Ribosomal protein L11	Rpl11	-1.035	6.91E-04	-0.877	2.57E-03
P27321	Calpastatin	Cast	-1.038	2.79E-03	-0.582	2.09E-01
A0A8I6AUS6	Purkinje cell protein 4-like 1	Pcp4l1	-1.046	1.41E-05	-0.989	1.78E-04
B0BN81	Ribosomal protein S5	Rps5	-1.055	4.25E-03	-0.950	9.39E-03
A0A8I5Y758	CDC42 effector protein (Rho GTPase binding) 4, isoform CRA_a	Cdc42ep4	-1.055	4.17E-04	-0.970	6.92E-04
A0A8L2R0X2	Fibulin 5	Fbln5	-1.069	1.93E-03	-0.673	4.03E-02
Q5D059	Heterogeneous nuclear ribonucleoprotein K	Hnrnpk	-1.087	2.42E-04	-0.994	7.74E-04
Q6MG48	Proline rich coiled-coil 2A	Prrc2a	-1.093	5.21E-04	-1.055	5.53E-04
P52944	PDZ and LIM domain protein 1	Pdlim5	-1.104	2.34E-04	-0.935	1.00E-03
A0A8I6GF21	Coiled-coil domain containing 50	Ccdc50	-1.104	1.26E-05	-1.047	1.50E-04
B0BMS8	Myosin regulatory light polypeptide 9	Myl9	-1.112	4.14E-04	-0.993	3.51E-03
D4AB03	Family with sequence similarity 120, member A	Fam120a	-1.115	2.37E-05	-0.900	2.34E-04

UniProt ID	Protein name	Protein ID	Medium vs. Sham		High vs. Sham	
			Log2(FC)[1]	FDR[2]	Log2(FC)	FDR
F7FIS4	Tropomyosin 1	Tpi1	-1.139	4.46E-03	-1.354	9.28E-04
P62076	Translocase of inner mitochondrial membrane 13	Timm13	-1.174	1.12E-04	-1.088	1.39E-04
A0A8I6AFQ2	Tight junction protein 1	Tjp1	-1.175	8.71E-05	-1.180	1.87E-04
O88797	Disabled homolog 2	Dab2	-1.179	8.86E-05	-1.138	3.10E-04
A6J8U6	Basal cell adhesion molecule, isoform CRA_a	Bcam	-1.187	2.90E-05	-1.135	2.17E-04
A6HEB4	Ribosomal protein L30	Rpl30	-1.198	3.93E-06	-0.914	4.84E-04
F1LW91	Nuclear mitotic apparatus protein 1	Numa1	-1.210	7.52E-04	-1.102	2.61E-03
P02771	Albumin	Alb	-1.232	1.03E-02	-0.862	1.00E-01
Q923W4	Hepatoma-derived growth factor-related protein 3	Hdgfl3	-1.251	9.72E-05	-0.972	5.68E-03
A0A8L2QEH3	Ribosomal protein S19	Rps19	-1.256	1.88E-05	-1.101	5.34E-04
A0A0G2K4X8	S-phase kinase-associated protein 1	Skp1	-1.266	1.18E-05	-1.188	8.21E-04
A6IF31	Serine protease 1	Prss1	-1.272	2.92E-04	-1.009	4.48E-03
A0A8I6A790	Chloride intracellular channel protein 1	Clic1	-1.283	4.14E-04	-1.206	2.69E-03
P32038	Complement factor D	Cfd	-1.284	1.73E-05	-1.073	8.16E-04
A6KD00	Heterogeneous nuclear	Hnrnpa1	-1.295	3.78E-04	-1.365	4.27E-04

UniProt ID	Protein name	Protein ID	Medium vs. Sham		High vs. Sham	
			Log2(FC)[1]	FDR[2]	Log2(FC)	FDR
	ribonucleoprotein A1					
A0A8I6GFT9	Formin binding protein 1	Fnbp1	-1.318	4.01E-05	-1.036	5.27E-04
A6I828	Lymphatic vessel endothelial hyaluronan receptor 1	Lyve1	-1.319	1.19E-05	-0.999	9.47E-03
A6KDU1	Thioredoxin 1	Txn1	-1.331	4.07E-05	-1.268	4.77E-04
A6KSD6	RCG42490, isoform CRA_b	RCG42490	-1.349	2.53E-04	-1.228	4.59E-04
A6JXV7	Dynein light intermediate chain	Dync1li2	-1.381	1.69E-04	-1.279	4.84E-04
A0A8I5ZMI8	Keratin, type II cytoskeletal 6A-like	LOC120093742	-1.387	1.37E-03	-1.376	1.86E-03
P18211	Rano class II histocompatibility antigen, D-1 beta chain	RT1-Db1	-1.423	2.34E-04	-1.274	4.73E-04
A0A8I5ZLU6	Afadin, adherens junction formation factor	Afdn	-1.434	1.02E-04	-1.265	7.30E-04
A0A8I6G597	Paralemmin 1	Palm	-1.463	1.20E-05	-1.442	1.01E-04
A6JQC5	Spectrin beta chain 1	Sptbn1	-1.464	1.39E-05	-1.310	6.46E-05
A0A8I5ZVN9	Ribosomal protein S11	Rps11	-1.478	7.56E-06	-1.371	6.52E-05
D4A2F2	Par-6 family cell polarity regulator beta	Pard6b	-1.489	2.52E-05	-1.089	1.05E-02
A6KAN9	MacroH2A.1 histone	Macroh2a1	-1.493	1.59E-06	-1.344	2.59E-05
A0A8I6ASE1	Antioxidant 1 cooper chaperone	Atox1	-1.507	9.77E-07	-1.242	1.16E-03

UniProt ID	Protein name	Protein ID	Medium vs. Sham		High vs. Sham	
			Log2(FC)[1]	FDR[2]	Log2(FC)	FDR
Q63575	Trans golgi network (TGN) specific integral membrane protein 41	Tgn41	-1.543	1.22E-05	-1.345	2.14E-03
P04166	Cytochrome b5 type B	Cyb5b	-1.546	4.98E-06	-1.408	6.11E-05
P62634	CCHC-type zinc finger nucleic acid binding protein	Cnbp	-1.546	5.55E-07	-1.401	2.25E-04
A6K8P9	Cold-inducible RNA-binding protein	Cirbp	-1.550	3.88E-05	-1.267	3.94E-03
A6KSI5	PYM homolog 1, exon junction complex associated factor	Pym1	-1.553	2.42E-06	-1.430	3.52E-05
A0A8I5Y0Q3	Family with sequence similarity 107, member B	Fam107b	-1.568	5.59E-05	-1.415	2.34E-04
A6JMG3	STING ER exit protein 1	Steep1	-1.590	4.38E-05	-1.160	2.43E-03
O35796	Complement component 1 Q subcomponent-binding protein, mitochondrial	C1qbp	-1.603	1.88E-04	-1.242	1.83E-02
A0A8I6G5X8	Heterogeneous nuclear ribonucleoprotein H1	Hnrnph1	-1.624	2.48E-05	-1.274	3.51E-03
A6K4W2	CGG triplet repeat binding protein 1	Cggbp1	-1.633	9.74E-07	-1.565	1.38E-05
A6HRN5	Alpha-1-B glycoprotein, isoform CRA_b	A1bg	-1.640	2.02E-05	-1.645	2.34E-04

UniProt ID	Protein name	Protein ID	Medium vs. Sham		High vs. Sham	
			Log2(FC)[1]	FDR[2]	Log2(FC)	FDR
A0A8I6AKF5	Synaptopodin	Synpo	-1.658	3.05E-06	-1.506	1.82E-05
F7F635	Alpha hemoglobin stabilizing protein	Ahsp	-1.663	6.26E-04	-1.754	4.45E-04
A6JMF3	Progesterone receptor membrane component 1	Pgrmc1	-1.681	1.14E-05	-1.596	5.83E-05
B0K013	Cytochrome c oxidase assembly factor 4	Coa4	-1.706	6.38E-06	-1.373	3.79E-04
A0A8L2QHS0	Ras interacting protein 1	Rasip1	-1.708	2.25E-07	-1.548	1.95E-05
Q5BKC3	Parkinsonism associated deglycase	Park7	-1.725	3.96E-06	-1.552	5.88E-05
Q9UFG5	Chromosome 19 open reading frame 25	C19orf25	-1.742	9.48E-06	-1.568	2.67E-04
G3V843	Coagulation factor II, thrombin	F2	-1.746	1.43E-06	-1.633	2.33E-05
A0A8L2UMW7	Parvalbumin	Pvalb	-1.759	1.33E-05	-1.26	1.12E-02
A0A8L2QE45	Craniofacial development protein 1	Cfdp1	-1.836	3.15E-07	-1.550	6.34E-05
A6JJQ5	Ribosomal protein L10A	Rpl10a	-1.845	2.06E-06	-1.695	2.58E-04
A0A8I6A5G9	Ganglioside GM2 activator	Gm2a	-1.895	1.98E-07	-1.704	3.38E-05
D3ZGK7	Carboxylic ester hydrolase 1C	Ces1c	-1.974	3.29E-04	-1.858	1.90E-03
A0A8I6AEU	Ribosomal protein S8	Rps8	-2.012	2.60E-06	-1.749	2.56E-04
D4A2W1	Ectonucleotide pyrophosphate/phosphodiesterase 4	Enpp4	-2.016	3.81E-05	-1.692	3.16E-03

UniProt ID	Protein name	Protein ID	Medium vs. Sham		High vs. Sham	
			Log2(FC)[1]	FDR[2]	Log2(FC)	FDR
A6I5T3	Integrin alpha 1, isoform CRA_b	Itga1	-2.020	9.99E-07	-1.772	6.34E-05
Q63610	Tropomyosin 3	Tpm3	-2.052	3.53E-06	-2.178	4.30E-05
A0A0G2JZ52	Heterogeneous nuclear ribonucleoprotein U	Hnrnpu	-2.082	1.40E-08	-1.856	2.45E-05
A6JHQ8	STE20 like kinase	Slk	-2.148	1.05E-07	-1.945	2.59E-05
A0A8I5ZVI9	RAN binding protein 3	Ranbp3	-2.15	2.17E-08	-1.842	1.68E-05
A0A0G2K3Z9	Peroxiredoxin-1	Prdx1l1	-2.194	6.61E-08	-2.221	2.16E-04
A6JS43	Histidine-rich glycoprotein	Hrg	-2.272	8.77E-07	-1.988	1.05E-04
A0A8I6AHB3	Lysosomal-associated membrane protein 2	Lamp2	-2.281	3.56E-07	-1.781	1.71E-04
A0A0G2K6Y9	Syntaxin 7	Stx7	-2.373	5.14E-08	-2.067	3.84E-05
Q02356	AMP deaminase 2	Ampd2	-2.422	2.17E-08	-2.090	1.10E-04
A6HZZ1	Secretoglobin family 1A member 1	Scgb1a1	-2.548	2.42E-06	-2.215	6.34E-05
A0A8I6AMC9	C3 and PZP-like, alpha-2-macroglobulin domain containing 8	Cpamd8	-2.569	5.36E-06	-2.645	3.34E-04
P05942	S100 calcium binding protein A4	S100a4	-2.57	1.60E-06	-2.303	1.87E-04
D3ZV50	Ribosomal protein S91 like 1	Rps9l1	-2.640	5.41E-08	-2.429	3.15E-05

UniProt ID	Protein name	Protein ID	Medium vs. Sham		High vs. Sham	
			Log2(FC)[1]	FDR[2]	Log2(FC)	FDR
A0A8L2PYP2	C-reactive protein	Crp	-2.802	1.05E-07	-2.566	3.15E-05
A0A0G2K151	Apolipoprotein E	Apoe	-2.840	6.33E-08	-2.799	1.20E-05
A0A8I6GID	Integrin subunit alpha 8	Itga8	-2.944	1.02E-09	-2.616	6.34E-05
F7FL28	Napsin A aspartic peptidase	Napsa	-2.966	2.07E-08	-2.423	8.79E-04
A6I4B3	C-type lectin domain family 3, member G	Clec3b	-3.090	1.34E-07	-2.526	7.91E-04
D4A636	IKAROS family zinc finger 4	Ikzf4	-3.285	3.45E-08	-2.725	5.06E-04
A0A8I6A7A5	Ribosomal protein S24	Rps24	-3.436	4.36E-09	-2.773	7.12E-04
Q6AYD4	Endothelial cell-selective adhesion molecule	Esam	-3.546	1.59E-10	-3.081	7.67E-05
A0A8L2QDS2	Glutathione S-transferase mu 2	Gstm2	-4.052	4.68E-11	-3.397	4.91E-04
Q6P790	Ribosomal protein L6	Rpl6	-4.599	1.88E-10	-3.994	8.88E-05
A6J2G7	Ribosomal protein S26	Rps26	-4.631	3.67E-11	-3.607	1.51E-03
A6JS59	Transformer 2 beta homolog	Tra2b	-4.653	3.04E-10	-3.813	2.74E-04
F1M9N4	Muscleblind-like splicing regulator 1	Mbnl1	-6.625	1.51E-12	-5.447	3.22E-04

[1]Log2 of fold-change of ±1.0

[2]False discovery rate represents the adjusted *P*-value of 0.05

Table S12. Principal component analysis loadings for proteomics.

**Sample**[1]	**PC1**	**PC2**
Sham 91	-16.7986	-1.15781
Sham 92	-16.8728	16.10833
Sham 94	-17.0653	3.601158
Sham 108	-17.3898	5.002726
Sham 109	-16.8439	-2.55159
Low 95	-16.6137	-3.8012
Low 96	-16.5873	-6.32854
Low 97	-16.9655	-3.46265
Low 112	-16.0680	-5.74951
Medium 99	15.39682	-8.57032
Medium 101	17.08286	3.339122
Medium 114	16.60709	1.349116
Medium 115	16.38472	5.047266
Medium 117	16.70616	-1.83666
High 102	1.557952	-3.47793
High 103	17.4508	-0.78452
High 104	16.99784	1.83938
High 105	16.05253	-2.81042
High 119	16.96811	4.24407

[1]4-OH-PCB52 polymeric implant exposure groups: Sham 0%; Low 1%; Medium 5%; High 10%

Table S13. Summary of RNA-sequencing read counts and quality control metrics for lung transcriptome libraries.

Sample[1]	Raw reads[2]	Clean Reads[3]	Raw data[4]	Effective[5] (%)	Error[6] (%)	Q20[7] (%)	Q30[8] (%)	GC[9] (%)
Sham 91	41169072	38011014	6.2	92.33	0.01	97.42	93.41	45.59
Sham 92	41647210	39402368	6.2	94.61	0.01	97.33	93.20	49.10
Sham 94	49719848	45794734	7.5	92.11	0.01	97.09	92.88	49.01
Sham 108	41627178	38007216	6.2	91.30	0.01	97.33	93.33	45.19
Sham 109	45667174	42097212	6.9	92.18	0.01	97.20	92.90	47.49
Low 96	47928012	44946308	7.2	93.78	0.01	98.61	96.01	45.30
Low 97	50244830	45681882	7.5	90.92	0.01	97.23	93.05	46.82
Low 112	42998536	40542644	6.4	94.29	0.01	97.38	93.34	47.31
Medium 99	48114388	44014372	7.2	91.48	0.01	97.27	93.25	48.27
Medium 101	52881654	47260312	7.9	89.37	0.01	97.79	94.13	47.01
Medium 114	41305566	36979434	6.2	89.53	0.01	97.77	94.15	47.44
Medium 115	43576058	37385572	6.5	85.79	0.01	97.59	93.89	47.44
Medium 117	41105738	37722230	6.2	91.77	0.01	97.22	93.09	47.63
High 102	46566600	41828010	7.0	89.82	0.01	97.92	94.51	48.82
High 103	46024854	40369526	6.9	87.71	0.01	97.81	94.33	48.81
High 104	47759008	42798216	7.2	89.61	0.01	97.92	94.48	49.10
High 105	41730368	36906546	6.3	88.44	0.01	97.71	94.02	46.91
High 119	45657272	41975272	6.8	91.94	0.01	97.24	93.09	46.24

[1]4-OH-PCB52 polymeric implant exposure groups: Sham 0%; Low 1%; Medium 5%; High 10%
[2]The total number of sequenced reads before any filtering
[3]The total number of reads that remain after quality control filtering
[4]The total size of sequencing data; calculated from raw reads x read length
[5]Percentage of clean reads relative to raw reads: effective (%) = (clean reads / raw reads) x 100

[6]The base-calling error rate of the sequencing data; calculated from Phred quality scores
[7]Percentage of bases with Phred quality score ≥ 20, 1% error rate (99% inferred base call accuracy)
[8]Percentage of bases with Phred quality score ≥ 30, 0.1% error rate (99.9% inferred base call accuracy)
[9]Percentage of guanine (G) and cytosine (C) bases in the reads

Table S14. Gene enrichment pathways associated with lung transcriptomics.

Pathway	activated (+) or suppressed (-); FDR		
	**Low**	**Medium**	**High**
Cytoplasmic translation	(+) 9.26E-09	-	(-) 0.00165
Import across plasma membrane	(-) 1.55E-05	-	-
Modulation of chemical synaptic transmission	(-) 2.44E-05	(-) 0.00032	(-) 0.02860
Regulation of trans-synaptic signaling	(-) 2.44E-05	(-) 0.00032	(-) 0.02860
Immune response-regulating cell surface receptor signaling pathway	(-) 0.00037	(-) 0.01538	(-) 0.00037
Endodermal cell fate commitment	(+) 0.00042	-	-
Sacromere organization	(-) 0.00149	-	(-) 8.97E-06
Plasma membrane fusion	(-) 0.00394	(+) 4.94E-06	(+) 0.02049
Olefinic compound metabolic process	(-) 0.01479	(-) 1.78E-05	-
Primary alcohol metabolic process	(-) 0.04277	(-) 2.33E-05	-
Cell projection morphogenesis	(-) 0.04898	(-) 2.89E-05	-
Cilium assembly	-	(+) 1.24E-06	(-) 0.00049
Cilium movement	-	(+) 1.60E-06	(-) 0.00036
Axoneme assembly	-	(+) 8.06E-05	(-) 2.13E-05
Microtubule bundle formation	-	(+) 0.00190	(-) 9.03E-06
Mesenchyme development	-	-	(+) 1.22E-05
Glycosylation	-	-	(+) 1.68E-05
Stem cell development	-	-	(+) 1.81E-05

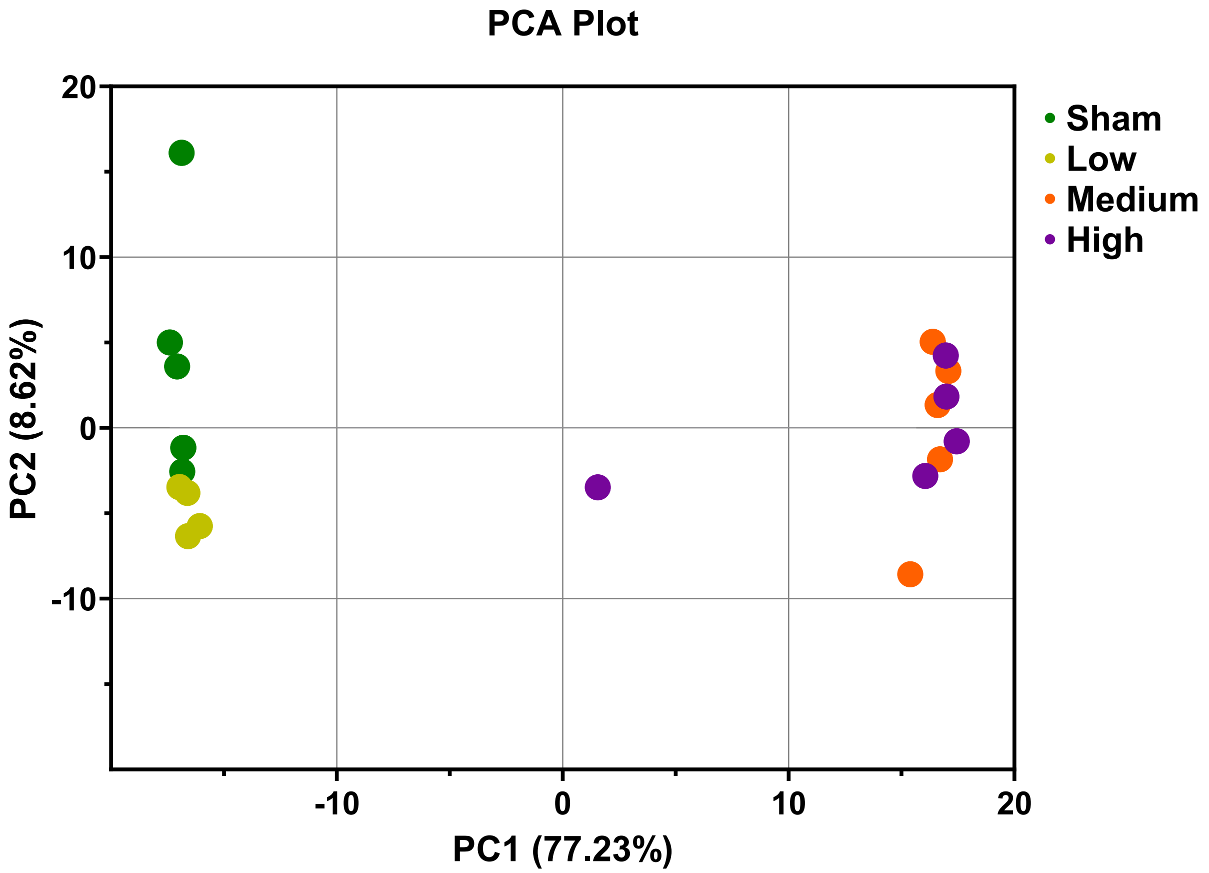

Figure S1. Principal component analysis (PCA) plot of lung proteomic samples. Each colored circle indicates different exposure groups of 4-OH-PCB52 polymeric implant exposure: green = sham control 0%; yellow = low 1%; orange = medium 5%; purple = high 10%. PC1 represents 77.23% of total variance, and PC2 represents 8.62%. Values were generated with Amica and illustrated in GraphPad Prism.

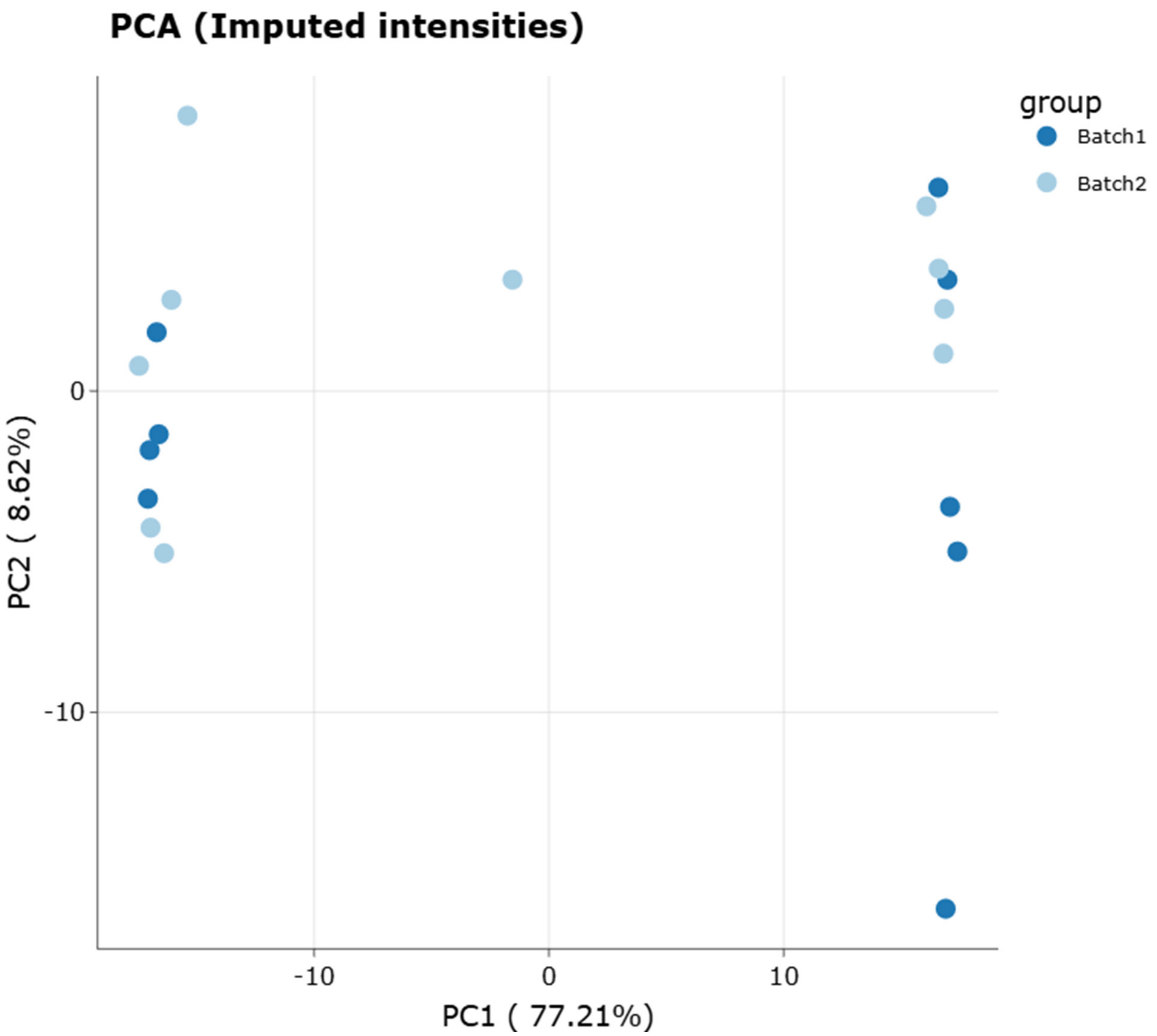

Figure S2. Principal component analysis (PCA) of normalized proteomic data across TMT batches. PCA of normalized protein abundance data following pooled master-control-based normalization. Samples from Batch 1 (dark blue) and Batch 2 (light blue) showed no distinct batch-specific clustering, indicating minimal residual inter-batch technical variation.

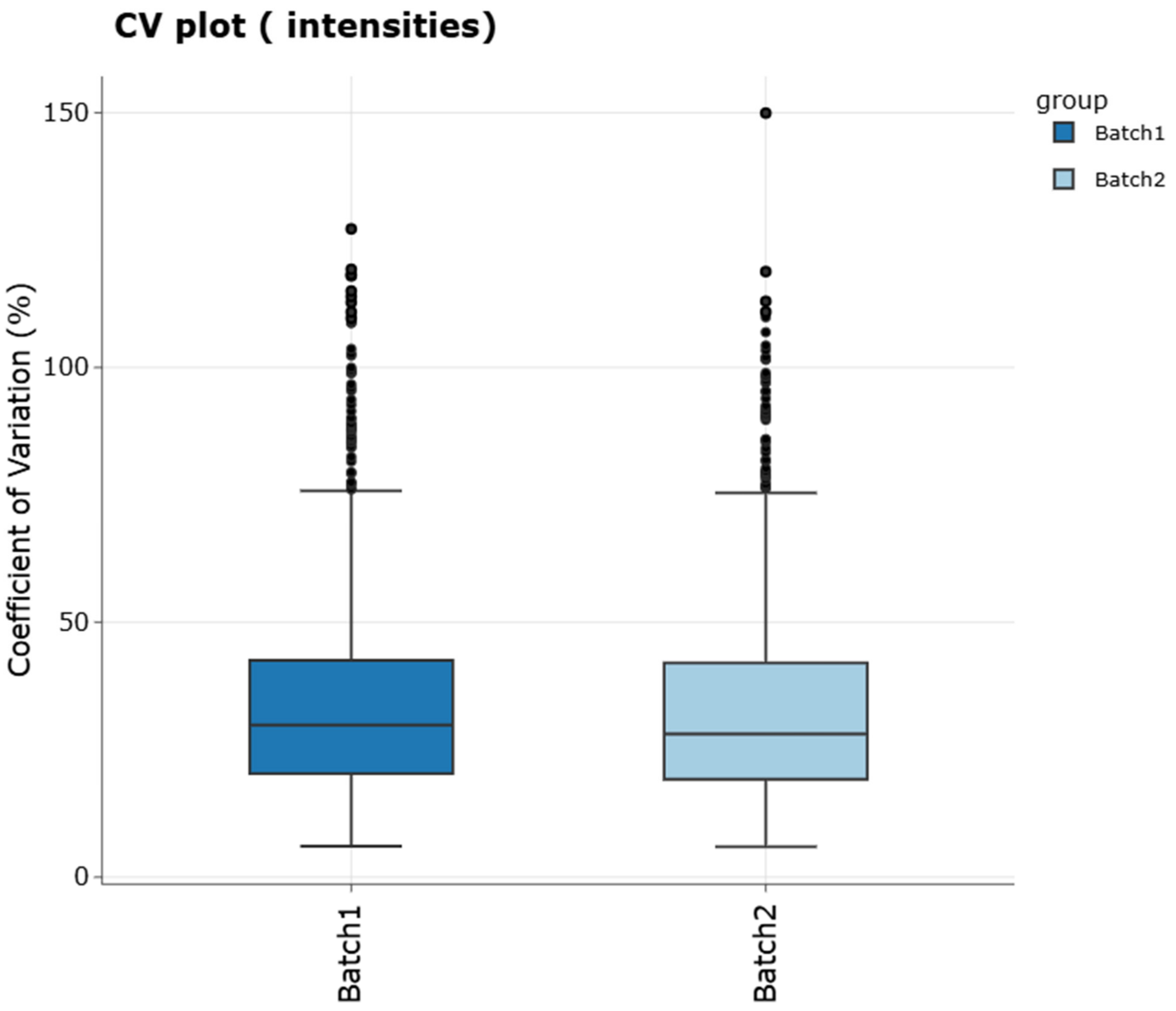

Figure S3. Coefficient of variation (CV) distributions across TMT batches. Boxplots showing the distribution of protein intensity CVs for Batch 1 and Batch 2 following normalization. Comparable CV distributions between batches indicate similar levels of variability and support effective reduction of inter-batch technical variation.

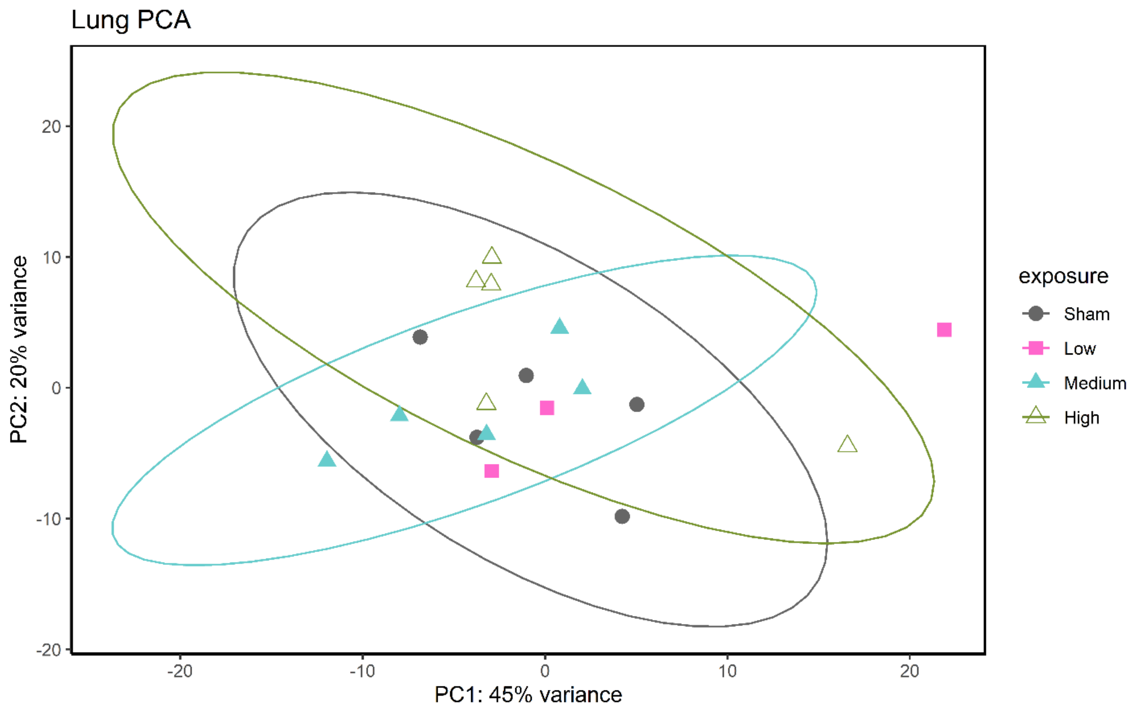

Figure S4. Principal component analysis (PCA) of lung transcriptomic profiles. Exposure groups are represented by a gray circle (sham 0%); pink square (low 1%); turquoise triangle (medium 5%); and green transparent triangle (high 10%). PC1 represents 45% of total variance, and PC2 represents 20%. The plots were generated using ggplot (v3.4.1) in R (v4.5.0) with R Studio (2025.08.12)

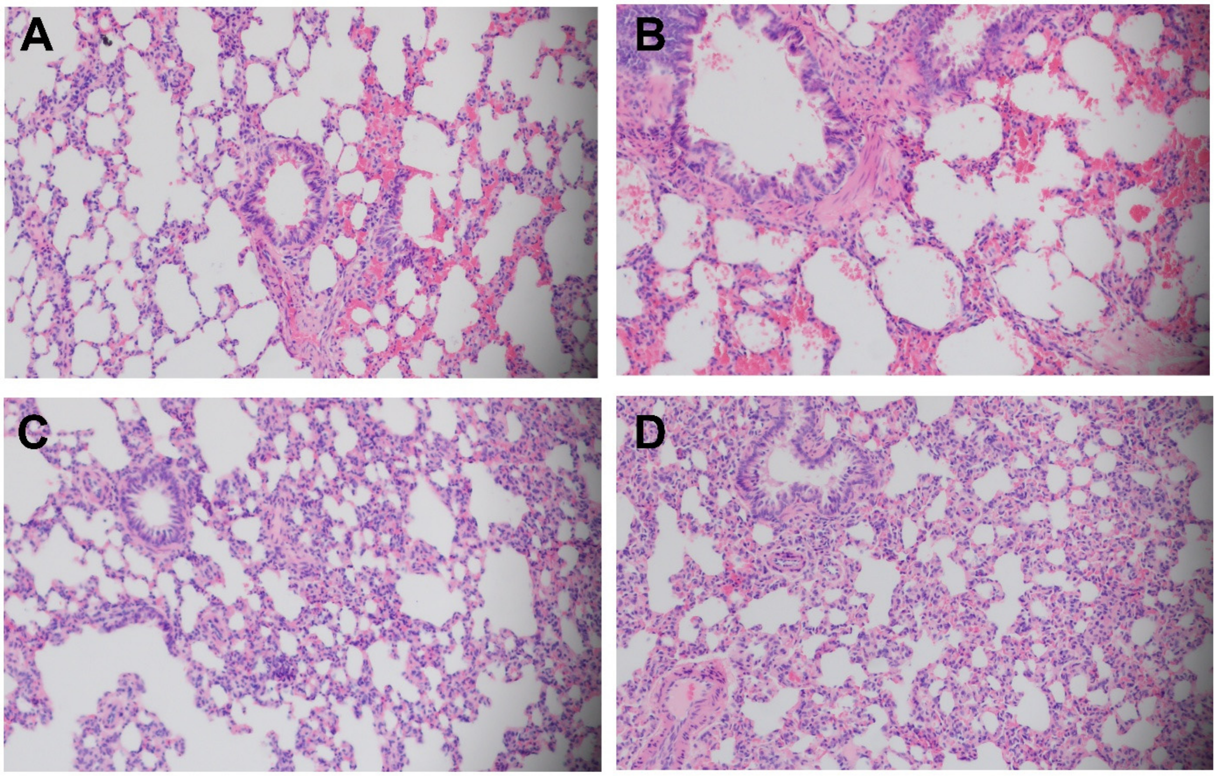

Figure S5. Representative images for the hematoxylin and eosin (H&E) staining of the lung histopathology in female PND 56 rats. (A) sham control, (B) low 1%, (C) medium 5%, (D) high 10% dose exposure. Hematoxylin = nuclei; eosin = cytoplasmic and extracellular matrix protein
